# Job quality and fertility intentions among Chinese migrant workers: the role of traditional fertility beliefs

**DOI:** 10.3389/fpsyg.2026.1739790

**Published:** 2026-06-17

**Authors:** Huihui Liu, Augustine Adira

**Affiliations:** College of Economics and Management, Southwest University, Chongqing, China

**Keywords:** China, job quality, migrant workers, short-term fertility intention, traditional fertility beliefs, value of children

## Abstract

Under conditions of low fertility, the association between overall working conditions and fertility intention, and the extent to which this association is accompanied by changes in traditional fertility beliefs, remain insufficiently understood, particularly among Chinese migrant workers, a population situated at the intersection of traditional cultural norms and the modern labor market. Using Value of Children theory as the core framework, and incorporating insights from Conservation of Resources theory and social identity theory, this study examines the association between job quality and short-term fertility intention among Chinese migrant workers and explores the potential role of traditional fertility beliefs in this association. Using data on 2,119 married migrant workers of childbearing age from the 2020 China Family Panel Studies (CFPS), this study constructs a composite job quality index encompassing six dimensions: wage income, job stability, work intensity, welfare protection, career development prospects, and job satisfaction, and employs a two-level mixed-effects logistic regression model for analysis. The results indicate that higher job quality is significantly associated with lower short-term fertility intention. Parity-stratified analysis reveals that this association is primarily concentrated among one-child families facing the decision of whether to have a second child and is not significant among families with two or more children. At the mechanism level, higher job quality is significantly associated with weaker endorsement of the “continuing the family lineage” belief in the full sample, whereas the association between higher job quality and weaker endorsement of the “raising children for old-age support” belief is significant only among migrant workers without male children. Furthermore, the negative association between job quality and short-term fertility intention is more pronounced among male migrant workers, whereas it is not significant among female migrant workers. By integrating Conservation of Resources theory into a Value of Children framework, this study provides empirical evidence for understanding the associations among employment conditions, traditional fertility beliefs, and fertility intention. The findings further suggest that these associations are conditional and context-dependent rather than universally applicable.

## 1 Introduction

Fertility intentions reflect planful judgments about future childbearing that are typically shaped by the combined influence of material circumstances, cultural belief systems, individual values, and broader social-structural contexts (Ajzen, 1991; Ajzen and Klobas, 2013; Bachrach and Morgan, 2013; Fishbein and Ajzen, 2010; Kohler, 2001). Against the backdrop of the global decline in fertility rates, understanding the mechanisms through which fertility intentions are formed has become an important topic of shared concern in both social psychology and population research. In China, although the government has successively introduced pro-natalist policies such as the universal two-child and three-child policies, the national birth rate has continued to decline. This makes the formation of fertility intentions among key social groups, and variation across subpopulations, an issue warranting further investigation. Migrant workers are a group that deserves particular attention in this regard: they typically hold agricultural hukou (China’s household registration system that classifies citizens as rural or urban residents) but are primarily engaged in non-agricultural labor. According to the 2020 China Population Development Social Survey, even with relaxed fertility policies, the average ideal and expected numbers of children for agricultural hukou holders are 1.64 and 1.65, respectively, both of which are below replacement level (Fudan University School of Social Development and Public Policy, 2020). As a population that has traditionally exhibited relatively high fertility, yet whose fertility intentions have also become increasingly salient under current low-fertility conditions, migrant workers provide a particularly meaningful setting for examining how employment conditions are linked to fertility intentions.

Migrant workers play a critical role in China’s socio-economic structure. As of 2023, China’s migrant worker population was approximately 297.53 million, with male and female workers comprising about 62.7 and 37.3%, respectively (National Bureau of Statistics of China, 2024). This large labor force not only drives economic growth but also tends to experience relatively low job quality. Due to limited skills, migrant workers are predominantly concentrated in labor-intensive industries such as manufacturing, construction, wholesale and retail trade, and services, with a high proportion engaged in informal employment. This employment structure subjects them to multiple constraints, including relatively low wages, poor job stability, high work intensity, low social insurance coverage, and limited career development opportunities (Chan, 2010; Keung Wong et al., 2007; Lee, 2012; Shi, 2008; Wang and Fan, 2012; Zhang et al., 2016). At the same time, this population occupies a transitional space between traditional rural culture and the modern urban labor market, and their fertility decisions are embedded in the tension between traditional values and modern economic realities (Myerson et al., 2010; Yu and Liang, 2022). In recent years, improving the employment quality of migrant workers has also gradually become an important focus of relevant policy efforts.

In this context, examining the association between migrant workers’ job quality and their fertility intentions can offer a valuable perspective for understanding how social-structural conditions are linked to belief systems and behavioral intentions, a core question in social psychology. Recent studies increasingly highlight the association between employment-related factors and fertility intentions, showing that factors such as unemployment (Busetta et al., 2019; Özcan et al., 2010; Pailhé and Solaz, 2012; Raymo and Shibata, 2017; Schmitt, 2012), job instability (Alderotti et al., 2021; Gatta et al., 2022; Hanappi et al., 2017; Modena and Sabatini, 2012; Modena et al., 2014; Vignoli et al., 2020a), job security (Lopes, 2020; Owoo and Lambon-Quayefio, 2022), contract types (Kreyenfeld et al., 2012; Vignoli et al., 2020b), perceptions or expectations regarding employment conditions (Begall and Mills, 2011; Gatta et al., 2022; Hanappi and Buber-Ennser, 2017; Hanappi et al., 2016), and work–family conflict (Call et al., 2008; Molina, 2021; Shreffler et al., 2010; Yu and Kuo, 2017) are associated with fertility intentions to varying degrees. However, most of this research has focused on European countries, and few studies have systematically evaluated how overall working conditions, including wages, job stability, and welfare protection, relate to fertility intentions, or examined the psychological mechanisms through which employment conditions may be linked to fertility decisions through traditional cultural beliefs.

Given this background, the present study adopts a job quality perspective to examine its association with fertility intentions among migrant workers. The close association between job quality and individual well-being has been widely recognized (see Clark, 2015; Eurofound, 2012; Green, 2006; Holman, 2013; de Muñoz Bustillo et al., 2011a, 2011b; OECD, 2013). For migrant workers, non-agricultural employment serves not only as the primary channel for acquiring economic resources (People’s Daily, 2015), but also as an important source of social recognition, identity affirmation, and a sense of dignity. In this regard, job quality, understood as the objective resource conditions and subjective well-being experiences individuals derive from work, has important implications for migrant workers’ overall life circumstances and may also be linked to their fertility intentions (Matsuo and Matthijs, 2016; Neyer et al., 2013; Novelli et al., 2021).

With respect to the theoretical framework, this study adopts the Value of Children (VOC) theory (Hoffman and Hoffman, 1973; Nauck, 2005, 2007) as the core framework for understanding fertility decisions. VOC theory holds that fertility intentions depend on individuals’ subjective assessments of the various values children may provide, including economic-utilitarian value, social recognition value, and emotional value, and that the composition of these values changes systematically across stages of socioeconomic development. Building on this foundation, the present study introduces Conservation of Resources (COR) theory (Hobfoll, 1989, 2002) as a key mechanism for explaining how job quality may be linked to the assessment of traditional child values: as the alternative resources provided by employment become increasingly abundant, the relative importance of the functional roles traditionally fulfilled by children may decline accordingly. Regarding the social recognition dimension embedded in the “continuing the family lineage” belief, a social identity perspective is further incorporated in subsequent sections to elaborate on this mechanism. Based on this framework, the present study focuses on the associational patterns among job quality (measured through a composite job quality index), traditional fertility beliefs, and short-term fertility intentions. The study further examines the scope of applicability of these associations across different parity contexts, as well as variations across gender and household income level groups.

To systematically explore the above issues, the study focuses on the following three research questions:

1. Is job quality associated with the short-term fertility intentions of migrant workers? Does this association vary by current family size (parity)?
2. Is job quality associated with traditional beliefs such as “raising children for old-age support” and “continuing the family lineage”? Does this association differ depending on whether migrant workers have male children?
3. Does the association between job quality and short-term fertility intentions vary by gender and household income level?

By addressing these research questions, this study seeks to contribute to the existing literature in three respects. First, at the theoretical level, this study adopts the Value of Children theory as its core framework and integrates Conservation of Resources theory and social identity theory, proposing an analytical framework for understanding how job quality may be linked to fertility intentions through the assessment of traditional child values. This approach connects employment conditions research more closely with the child value perspective in fertility theory. Second, at the empirical level, unlike existing studies that predominantly focus on single employment variables, this study constructs a composite job quality index encompassing wage income, job stability, work intensity, welfare protection, career development prospects, and job satisfaction, and systematically examines the associational patterns among overall job quality, traditional fertility beliefs, and fertility intentions. Third, in terms of the study population and context, this study focuses on Chinese migrant workers, a group situated at the intersection of traditional cultural norms and modern labor markets, providing new empirical evidence for understanding the complex relationships among employment conditions, cultural beliefs, and fertility decisions in a transitional society.

## 2 Theoretical analysis and hypothesis development

### 2.1 Conceptualizing job quality

Core issues surrounding job quality, such as work design, work experience, and the structure of job rewards, have been systematically discussed in academic research since the early 1970s (Davis and Taylor, 1972; Hackman and Oldham, 1976; Kalleberg, 1977). In the early 2000s, as the European Union established “more and better jobs” as a strategic goal and promoted the development of multidimensional indicator systems (European Commission, 2001; European Council, 2000; European Foundation for the Improvement of Living and Working Conditions, 2002), job quality was further developed as a monitorable, comparable analytical framework for policy analysis. It should be emphasized that this multidimensional indicator system primarily serves the needs of policy governance and cross-national comparison. In academic research, however, approaches to constructing composite job quality indices—including dimension selection, weighting methods, and the integration of objective and subjective indicators—remain diverse, and no fully unified empirical standard has yet been established (European Commission, 2001).

Building on this foundation, the concept has evolved across different research traditions, giving rise to diverse interpretations and applications. Job quality is often understood as encompassing aspects of work that contribute to individual well-being, emphasizing the positive association between high-quality employment, quality of life, and economic performance (Cazes et al., 2015; Clark, 2015; Green, 2006; Holman, 2013; de Muñoz Bustillo et al., 2011a, 2011b; OECD 2013). In this study, job quality is defined as the composite of objective resource conditions and subjective work-related well-being experiences associated with migrant workers’ non-agricultural employment. In this context, well-being refers specifically to the material conditions and work-related experiences individuals derive from employment, rather than to quality of life in a broader sense. Although the academic literature has not reached consensus on a single comprehensive empirical indicator of job quality, most studies favor multidimensional measurement based on key aspects of work (European Commission, 2001). Consequently, job quality is widely recognized as a multidimensional concept (Dahl et al., 2009; Davoine et al., 2008; Fernández-Macías, 2012; Findlay et al., 2013; Green, 2006; Kalleberg, 2000, 2011; Martel and Dupuis, 2006), encompassing a range of objective characteristics, including labor compensation, working conditions, job stability, work intensity, social security, and career prospects (Anker et al., 2003; Bonnet et al., 2003; Howell and Kalleberg, 2019). At the subjective level, job quality is often captured through job satisfaction and individuals’ perceptions of work-related conditions (Begall and Mills, 2011; Brown et al., 2012; Clark, 2015; de Bustillo Llorente and Macias, 2005; Green, 2006; Hanappi and Buber-Ennser, 2017; Hanappi et al., 2016).

In light of this, the present study moves away from a unidimensional approach based on a single indicator and instead adopts a multidimensional analytical framework built around a composite index. Within this framework, we do not differentiate between specific high-quality and low-quality job types; rather, we focus on how the overall level of job quality is associated with migrant workers’ fertility intentions. This approach not only reflects the core concerns of job quality research but also helps capture the well-being conditions of migrant workers across diverse employment situations. The use of composite indices has become increasingly common in research on human well-being and social progress, and job quality research constitutes a closely related field (Anker et al., 2003; Boccuzzo and Gianecchini, 2015; Bonnet et al., 2003; Davoine et al., 2008; Leschke and Watt, 2008, 2014). In this study, we construct a composite index covering six dimensions: wage income, job stability, work intensity, welfare protection, career development prospects, and job satisfaction.

The selection of these six dimensions for constructing the composite job quality index is grounded in the following theoretical and empirical considerations. First, from the perspective of COR theory, job quality can be understood as a set of key well-being resources that workers are able to acquire, maintain, and use to buffer risks and improve their quality of life within the employment context (Hobfoll, 1989). Based on this framework, the six dimensions selected in this study collectively encompass the core types of well-being resources that migrant workers derive from non-agricultural employment. Wage income represents the most direct form of resource, constituting the material foundation for meeting basic living and consumption needs, and is typically regarded as a core dimension in multidimensional job quality measurement frameworks (Alpert et al., 2019; Cazes et al., 2015; Warhurst et al., 2017); however, relying on income as a single indicator is insufficient to fully explain the role of job quality in individual well-being (Esenaliev and Ferguson, 2019). Welfare protection (pension, medical, work injury, unemployment, and maternity insurance), as a core component of the social protection system, plays an important role in buffering income loss, reducing employment risks, and strengthening individuals’ capacity to cope with future uncertainties (Anderson and Pontusson, 2007; OECD, 2013), and is widely recognized as an important dimension of job quality (Cazes et al., 2015; Clark and Postel-Vinay, 2009; ILO, 2013). Job stability corresponds to the predictability of resource acquisition: stable employment relationships are associated with greater psychological security and lower job insecurity, thereby facilitating long-term planning (De Cuyper and De Witte, 2007; Greenhalgh and Rosenblatt, 1984; Plomp et al., 2019; Sverke et al., 2002). Work intensity reflects the expenditure of time and energy resources; within the framework of time allocation theory, excessively long working hours squeeze the time available for family life and increase physical and psychological burdens (Becker, 1965; Bianchi et al., 2006; Green, 2006; Kalleberg, 2011; Presser, 2003). Career development prospects embody the possibility of future resource gains, providing workers with expectations and goal orientation for improving their well-being (Dik et al., 2015, 2019; Gao and Smyth, 2011; Kalleberg, 2011; Redekopp and Huston, 2020). Job satisfaction, as a subjective evaluation dimension, reflects individuals’ overall perception of their work situation and their affective well-being, capturing experiential differences that objective indicators may fail to detect. It should be noted that satisfaction is not a summary indicator of job quality in itself; rather, it is more appropriately incorporated as a subjective well-being dimension within a multidimensional framework (Brown et al., 2012; Clark, 2015; Green, 2006). In sum, these six dimensions correspond, respectively, to material resources, institutional risk-buffering resources, stability resources, time and energy resources, developmental opportunity resources, and subjective well-being indicators, together constituting the broad set of well-being resources that employment may provide to migrant workers.

Second, from a methodological standpoint, these six dimensions can all be operationalized through measurable indicators available in the CFPS questionnaire data and are highly consistent with the core dimensions of mainstream international job quality frameworks (Cazes et al., 2015; Eurofound, 2012, 2014; ILO, 2013, 2022). For example, the OECD’s Job Quality Framework organizes its indicator system around three broad dimensions: earnings quality, labor market security, and quality of the working environment (Cazes et al., 2015); Eurofound’s European Working Conditions Survey systematically covers elements such as pay, working time, work-related stress, and skills development (Eurofound, 2012); and the ILO’s decent work indicator framework explicitly includes items such as adequate earnings, decent working time, stability and security of work, and social security (ILO, 2013). This measurement strategy helps ensure that the framework used in this study is both theoretically grounded and broadly comparable with existing research.

### 2.2 Theoretical framework: value of children, conservation of resources, and fertility intentions

#### 2.2.1 Value of children theory and fertility decision-making

Fertility intentions are not merely responses to economic costs; rather, they reflect individuals’ subjective assessments, formed within specific social contexts, of the values that children may provide. The Value of Children theory (VOC) posits that parental fertility decisions depend largely on the perceived values that children can offer, including economic-utilitarian value, social normative value, and emotional fulfillment value (Hoffman and Hoffman, 1973). Nauck (2005, 2007) further argued that the composition of child values is not fixed but undergoes systematic change across stages of socioeconomic development: in traditional agricultural societies, children’s economic value (e.g., household labor contribution, old-age security) and social recognition value (e.g., continuing the family lineage and fulfilling cultural responsibilities) occupy a dominant position; as modernization progresses and institutionalized social security systems are established, these functional values are gradually replaced, while the emotional value of children rises in relative importance. This theoretical perspective implies that changes in fertility intentions can be understood as the result of shifts in what parents expect to gain from children and how important those returns are perceived to be. With respect to the short-term fertility intentions examined in this study, when individuals perceive a decline in the traditional functional values of children, their short-term fertility intentions at a given reproductive stage may also decline accordingly.

#### 2.2.2 The contextual specificity of Chinese migrant workers

Applying Value of Children theory to Chinese migrant workers requires careful attention to the distinctive social context in which they are situated. Specifically, migrant workers’ fertility decisions are embedded in a transitional setting at the intersection of traditional rural culture and the modern urban labor market, a setting characterized by three salient features.

First, migrant workers remain deeply influenced by traditional fertility norms at the cultural level. Having grown up within the rural patriarchal family system centered on Confucian culture, traditional fertility norms remain deeply embedded in their value systems. In rural China, traditional fertility beliefs such as “more children, more blessings” are rooted in the economic utility of children for old-age support and the cultural responsibility of continuing the family lineage (Tang, 1995; Zhao, 1997). From an economic perspective, children are viewed as savings instruments and risk hedging tools, which is particularly important in an environment lacking effective social security systems (Feng, 2017; Mueller, 2013; Nugent, 1985; Wang, 2006). From a cultural perspective, continuing the family lineage is regarded as an important component of filial piety, and failure to fulfill this responsibility is considered unforgivable (Bedford and Yeh, 2019; Fan, 2006; Lee and Kuo, 2000). The maxim “Among the unfilial, having no descendants is the worst” not only emphasizes the importance of fulfilling this cultural responsibility but also occupies a central position in Confucian thought (Chan et al., 2002; Cole, 1998; Fan, 2006; Ho, 1996; Hwang, 2012; Kuo, 1998; Sarkissian, 2010). For many farmers, not having a son means being unable to carry on the family line, which is not only perceived as a failure toward one’s ancestors but also often invites negative social evaluations, occupying an important place in their belief systems, identity affirmation, and social recognition. Here, “social recognition value” does not refer generically to a sense of group belonging, but specifically to the social appraisal that individuals obtain through childbearing, particularly through bearing sons, by being regarded as qualified family members, fulfilling their familial responsibilities, and being seen as “complete adults.” These deeply rooted cultural beliefs emerged from a specific socioeconomic context: a self-sufficient agrarian economy based primarily on agricultural production, small-scale family-based farming operations, and a patrilineal and patriarchal family system centered on Confucian culture (Tang, 1995). In this context, childbearing served multiple functions, including increasing the labor force, realizing family property inheritance, ensuring parental old-age support, and continuing ancestral sacrifices, making the birth of sons extremely important (Hong et al., 1993; Kuo, 1998). However, for migrant workers who have long participated in the non-agricultural labor market, modern occupational experiences and urban lifestyles also continuously reshape their value judgments, such that traditional fertility norms do not persist in a singular, static manner but rather coexist with new life experiences. The tension between cultural norms and lived experience is precisely what the following two features describe.

Second, migrant workers are economically dependent primarily on non-agricultural employment. The mode of production for migrant workers has undergone a fundamental transformation: they rely primarily on non-agricultural employment for their livelihoods, with wage income becoming their core source of earnings (People’s Daily, 2015). This transformation in the mode of production means that the self-sufficient agricultural economic foundation upon which the aforementioned traditional fertility beliefs depended has been destabilized.

Third, the sources of welfare protection and social identity are undergoing a transfer. The economic security (old-age support) and identity value (family continuation and social recognition) that were traditionally obtained through children are now being partially assumed by work income, social insurance, and career development.

This overlapping state of traditional and modern convergence has resulted in contemporary migrant workers’ fertility beliefs exhibiting characteristics of mutual penetration between tradition and modernity (He, 2021; Liang et al., 2014), rendering the degree of dependence on traditional child values highly variable. In other words, traditional and modern elements coexist in migrant workers’ fertility beliefs, rather than replacing one another in a linear fashion. This variability also provides a valuable research setting for examining the associations among employment conditions, traditional child value assessments, and fertility intentions.

In the terminology of Value of Children theory, the two categories of traditional child values examined in this study can be summarized as follows: first, children’s economic security value, embodied in the “raising children for old-age support” belief, whereby children are viewed as an important source of old-age economic support and risk buffering; second, children’s social recognition value, embodied in the “continuing the family lineage” belief, whereby individuals seek to perpetuate the family bloodline, fulfill cultural responsibilities, and obtain social recognition through childbearing, particularly through bearing sons.

#### 2.2.3 Conservation of resources theory and the mechanism linking job quality to child value assessments

How, then, might employment conditions be linked to individuals’ assessments of these two categories of traditional child values? This study adopts Value of Children theory as the core framework for understanding fertility decisions and introduces Conservation of Resources theory (COR) as the key mechanism for explaining how job quality may reshape traditional child value assessments. COR theory posits that individuals are motivated to acquire, maintain, and protect valued resources, and that resource availability shapes belief systems, values, and behavioral intentions in important ways (Hobfoll, 1989, 2002). An important corollary of this theory is that the importance individuals attach to a particular resource is not fixed but adjusts with the availability of alternative resources (Hobfoll, 1989, 2002): when alternative resources become abundant, the relative importance of the original resource declines accordingly.

Within the framework of this study, job quality is understood as a comprehensive set of well-being resources that migrant workers acquire from non-agricultural employment. Here, “well-being” does not refer broadly to quality of life, but specifically denotes the objective resource conditions and subjective work experiences that individuals obtain through employment: the former includes income, welfare protection, and related material conditions, while the latter encompasses perceptions of and satisfaction with one’s work. Together, these two dimensions constitute the core content of job quality. As described in Section 2.1, this study measures job quality across six dimensions: wage income, job stability, work intensity, welfare protection, career development prospects, and job satisfaction. Chinese migrant workers commonly face low wages, long working hours, job instability, and limited opportunities for career advancement, all of which undermine their overall well-being (Chan, 2010; Keung Wong et al., 2007; Lee, 2012; Shi, 2008; Wang and Fan, 2012; Zhang et al., 2016). As the primary channel through which migrant workers acquire well-being resources, low job quality may trigger a dual deficit in material and psychological well-being, initiating the resource loss spiral described in COR theory (Hobfoll, 1989), increasing stress, and undermining overall well-being. Under such conditions, individuals are more likely to rely on traditional family-based security logic to interpret future risks and life arrangements, thereby maintaining stronger endorsement of both the economic security value and the social recognition value of children. When these resources are abundant, the well-being resources provided by employment partially substitute for the functions traditionally fulfilled by children, and the relative importance of traditional child values may decline accordingly, as individuals tend to invest resources in protecting and enhancing their existing work-related well-being. When individuals need to make fertility decisions in light of their current family circumstances and resource conditions, this resource-protective orientation is more likely to be associated with lower short-term fertility intentions.

**Figure 1:**
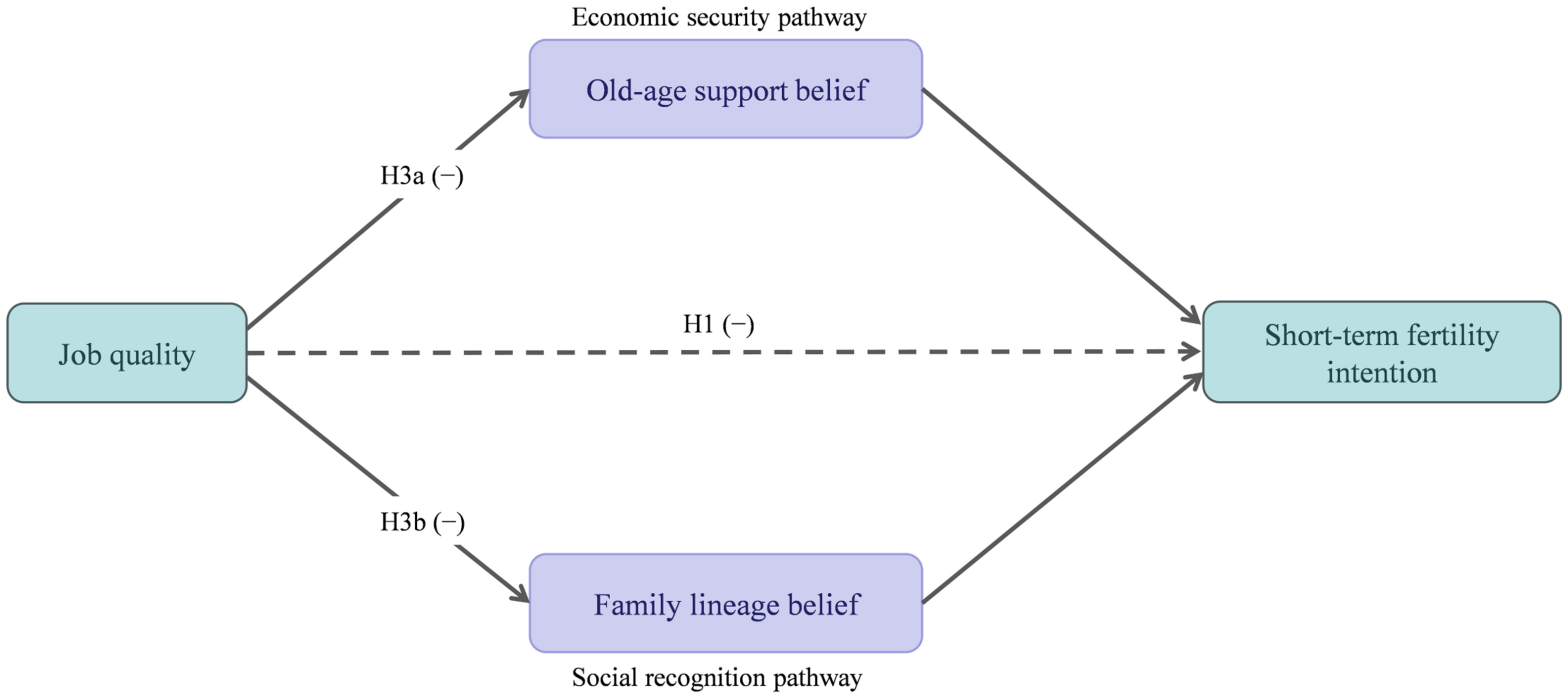
Theoretical framework and associational pathways.

In this process, changes in traditional fertility beliefs form the key evaluative link between job quality and fertility intentions. Specifically, this study proposes two associational pathways (see Figure 1).

Pathway 1: The Economic Security Pathway. Improvements in job quality provide migrant workers with more abundant economic and material resources as well as more comprehensive institutional protections (e.g., higher income, more stable employment, and broader social insurance coverage), which partially take over the functional role traditionally played by children in old-age security and risk buffering (Eurofound, 2014; Gallie, 2009; OECD, 2013). When individuals can obtain relatively adequate economic security through employment, their dependence on children as old-age security instruments decreases, and the relative importance of the “raising children for old-age support” belief weakens accordingly. This pathway follows the resource substitution logic of COR theory: the availability of alternative economic security resources diminishes the subjective importance of children’s economic security value.

Pathway 2: The Social Recognition Pathway. Unlike the instrumental logic underlying the “raising children for old-age support” belief, the weakening of the “continuing the family lineage” belief involves more complex psychological mechanisms. In traditional rural society, the “continuing the family lineage” belief served an important psychological well-being function: by bearing sons and perpetuating the family bloodline, individuals obtained social recognition, fulfilled cultural responsibilities, and avoided negative social evaluations. However, improvements in job quality not only enhance material conditions but also provide migrant workers with alternative pathways to social recognition and identity affirmation. Higher-quality employment, through occupational achievement, improved economic circumstances, and elevated social status, provides individuals with new identity resources and avenues for social recognition, partially substituting for the social approval traditionally acquired through bearing sons. Social identity theory offers a complementary theoretical explanation for this process: individuals construct positive self-evaluations through group membership and social comparison (Tajfel and Turner, 1979), and when occupational achievement becomes an accessible pathway for identity affirmation, the psychological need to obtain social recognition through bearing sons weakens accordingly. In this process, the identity affirmation and social recognition functions carried by the “continuing the family lineage” belief are partially substituted by the material resources, sense of dignity, and positive self-evaluations associated with high-quality employment, and the relative importance of this belief declines accordingly.

#### 2.2.4 Normative family size and contextual constraints on fertility decisions

The applicability of the above mechanisms is further shaped by an important contextual factor: normative family size. The focus here is not on individuals’ general preferences for lifetime ideal family size, but rather on their short-term fertility intentions as formed under the constraints of current family size and life-course stage. Fertility decisions are not solely based on economic considerations but also involve the satisfaction of emotional needs and conformity to social norms (Elster, 1998; Kagitcibasi and Ataca, 2005; McAllister et al., 2016). In contemporary China, social norms generally expect couples to have at least one child (Palmer, 1995; Santos and Harrell, 2016; Yu, 2022). According to the 2020 China Population Development Survey, only a minority (7.1%) believed that not having children was the ideal choice (Fudan University School of Social Development and Public Policy, 2020). This means that the first child carries extremely high emotional and social value, and its utility is difficult to substitute with employment resources.

However, once the first child is born and the basic expectation of parenthood is fulfilled, the marginal utility of subsequent births diminishes. Under current fertility norms, two children are widely regarded as the ideal family size (Guo, 2023; Lu et al., 2023). For one-child families, “whether to have a second child” represents an active decision oriented toward this normative benchmark, requiring the weighing of resource allocation against marginal returns. It is precisely at this decision point that the trade-off between the alternative resources provided by job quality and traditional child values is most likely to manifest: as the economic security and social recognition resources provided by employment become increasingly abundant, the motivation to pursue traditional child values through additional childbearing weakens. By contrast, for families with two or more children who have already met or exceeded this normative benchmark, subsequent childbearing is more likely to be shaped by individual preferences or special circumstances, and changes in job quality may be less likely to produce further significant associations.

Therefore, although this study focuses on short-term fertility intentions in a general sense, the formation of such intentions does not occur independently of existing family size and reproductive stage but is instead embedded in the specific context of family size and life-course stage. This reasoning is consistent with Nauck’s (2007) theoretical perspective: the assessment of child values is framed by specific social contexts and family conditions, and the decision-making weight it carries varies across different reproductive stages.

#### 2.2.5 Boundary conditions: heterogeneity in mechanism pathways and resource substitution capacity

The strength of the two associational pathways described above is unlikely to be uniform across all groups and may instead vary under several boundary conditions. This study examines these boundary conditions at two levels. The first concerns heterogeneity in the mechanism pathways themselves—specifically, whether the association between job quality and traditional fertility beliefs differs depending on whether individuals have fulfilled the core “reproductive task” prescribed by traditional patrilineal culture, that is, whether they have borne a son. The second concerns whether the substitution capacity of the well-being resources provided by job quality for traditional child values differs by gender.

The first level concerns the presence or absence of male children as a boundary condition for heterogeneity in the mechanism pathways. Within the two pathways described in Section 2.2.3, the strength of the association between job quality and traditional fertility beliefs may differ depending on whether migrant workers have already completed the “reproductive task” prescribed by traditional culture. In traditional patrilineal culture, filial piety is primarily seen as a duty of sons (Lee et al., 2009). After marriage, men are expected to support their parents, whereas women typically cease to bear this obligation upon marriage (Greenhalgh, 1985; Gruijters and Ermisch, 2019; Whyte and Xu, 2003). The patriarchal system also emphasizes the irreplaceable role of male children in continuing the family lineage (Zhang and Li, 2017; Zhao, 1997), making the birth of sons a primary goal for farming households (Arnold and Zhaoxiang, 1992; Greenhalgh and Li, 1995; Gruijters and Ermisch, 2019; Murphy et al., 2011; Wang, 2005). This means that the two categories of traditional child values, “raising children for old-age support” and “continuing the family lineage,” exhibit asymmetric fulfillment states along the dimension of whether a son has been born. For migrant workers who already have sons, both values have, at least in traditional cultural terms, largely been fulfilled, and the corresponding social recognition has also been largely realized. For migrant workers who do not yet have sons, both values remain unfulfilled, their subjective importance is therefore greater, and the cultural pressure attached to “continuing the family lineage” is stronger.

Based on the resource substitution logic of COR theory, when a category of traditional child value has not yet been realized and its subjective importance is relatively high, individuals are more likely to compare the newly gained well-being resources from job quality with the functions originally entrusted to children. The substitution effect of job quality is therefore more likely to emerge. For migrant workers without sons, when job quality improves, occupational achievement and improved economic circumstances serve as alternative security and identity resources, and their compensatory role becomes more apparent: the economic security, identity resources, and social recognition pathways provided by high-quality employment partially compensate for the security gap and lack of social recognition these workers experience in traditional culture due to not yet having borne a son. In contrast, migrant workers with sons have already fulfilled the cultural responsibility of continuing the family lineage and have obtained the corresponding social recognition. For them, improvements in job quality represent more of an accumulation of well-being than a substitute for traditional beliefs. The association between job quality and weakened traditional beliefs is therefore comparatively less pronounced. It should be emphasized that this hypothesis concerns heterogeneity in the mechanism pathways themselves, that is, whether the association between job quality and traditional fertility beliefs differs depending on the presence or absence of male children, rather than directly comparing the strength of the association between job quality and fertility intentions across groups.

The second level concerns gender as a boundary condition for heterogeneity in resource substitution capacity. Within the theoretical framework of this study, the association between job quality and fertility intentions is fundamentally mediated by the substitution of traditional child values by the well-being resources that job quality provides. However, this substitution capacity may not operate in the same way for men and women, because it is shaped by the gendered division of household labor and the unequal distribution of childbearing and caregiving costs. Fertility intentions derive from subjective assessments of child values, a logic that applies to all individuals. Yet in the Chinese context, men and women occupy markedly different structural positions when facing childbearing, and these differences affect whether the resources provided by job quality can be effectively translated into substitutes for traditional child values.

In the Chinese context, men are typically still expected to assume the primary economic responsibility for the family, and their social evaluation is more closely tied to occupational performance, earning capacity, and family provisioning ability. Therefore, when job quality improves, men are more readily able to obtain alternative security and identity resources through career development, income improvement, and social status enhancement, thereby reducing their dependence on the traditional values of children. In other words, for men, high-quality employment is more likely to substitute for some of the functions children traditionally served in relation to family responsibility, social evaluation, and future security. It is therefore more likely to prompt a reassessment of whether childbearing remains a necessary path for realizing self-worth and fulfilling family responsibilities.

In contrast, although women also benefit from high-quality employment in terms of resource gains, their childbearing-related costs are typically more direct and concentrated, primarily reflected in physical burdens, risks of career interruption, and childcare and housework responsibilities (Han, 2023; Ueno, 1990). Under conditions in which dual-role pressures remain salient, the resource gains from improved job quality are more easily offset by the anticipated costs of future childbearing and caregiving, and may not translate as effectively into substitutes for traditional child values as they do for men. Women’s fertility intentions are therefore more strongly constrained by the combined burden of childbearing, caregiving, and career interruption than by the resource improvements brought about by job quality alone. Accordingly, the capacity of resources provided by job quality to substitute for traditional child values may be stronger among men than among women, and the negative association between job quality and fertility intentions may therefore be more pronounced among male migrant workers.

Furthermore, household income level may also influence the strength of the association between job quality and fertility intentions. Based on COR theory and the law of diminishing marginal utility, families at different income levels may differ in the marginal gains they experience from changes in job quality and in the degree of resource substitution (Becker, 1981; Jones et al., 2008). High-income families already possess relatively abundant resources, and the marginal well-being gains from improved job quality are comparatively limited; low-income families, due to resource scarcity, may still view having more children as a means of increasing the family labor force or old-age security under traditional beliefs; middle-income families may occupy the range in which the marginal utility of well-being resources is most sensitive. However, this reasoning involves a complex interaction of multiple factors, including diminishing marginal utility, opportunity costs of childbearing, and the differential importance of traditional child values across income groups, making it difficult to derive a single, unambiguous prediction from the core theoretical framework. Therefore, household income differences are treated as part of an exploratory heterogeneity analysis rather than as the basis for a formal hypothesis.

Taken together, the above arguments suggest that job quality may be linked to migrant workers’ short-term fertility intentions through changes in the subjective assessment of traditional child values. The following section derives the specific research hypotheses on this basis.

### 2.3 Hypothesis development

#### 2.3.1 The overall association between job quality and short-term fertility intentions

When job quality is low, migrant workers are more likely to face deficits in material and psychological well-being resources, and the dominant position of traditional child values in their belief systems is therefore more likely to be maintained. When job quality is high, the alternative economic security, identity, and social recognition resources provided by employment become more abundant, and the relative importance of traditional child values may decline accordingly. Thus, the following hypothesis is proposed:

*Hypothesis 1:* Job quality is negatively associated with short-term fertility intentions among migrant workers.

#### 2.3.2 Parity differences and scope conditions for the association

In contemporary China, the first child typically carries strong emotional and social normative value, and these values are relatively difficult to substitute with employment resources. Once the first child is born and the basic expectation of parenthood is to some extent fulfilled, the marginal utility of subsequent births tends to decline, and whether to continue childbearing is more likely to become a practical decision requiring the weighing of resource allocation against marginal returns. By contrast, among families with two or more children, fertility intentions are generally lower, and the association between job quality and short-term fertility intentions may therefore be more limited. Thus, the following hypothesis is proposed:

*Hypothesis 2:* The negative association between job quality and short-term fertility intentions is more pronounced among one-child families.

#### 2.3.3 Traditional fertility beliefs as mechanism pathways

Along the economic security pathway, improvements in job quality provide migrant workers with alternative economic security resources, thereby reducing their dependence on children as instruments of old-age support. Along the social recognition pathway, improvements in job quality provide migrant workers with alternative resources for identity affirmation and social recognition, thereby weakening the need to obtain social recognition through bearing sons. Thus, the following hypotheses are proposed:

*Hypothesis 3a:* Higher job quality is associated with weaker endorsement of the “raising children for old-age support” belief among migrant workers.

*Hypothesis 3b:* Higher job quality is associated with weaker endorsement of the “continuing the family lineage” belief among migrant workers.

#### 2.3.4 Presence or absence of male children and heterogeneity in mechanism pathways

In traditional patrilineal culture, the two categories of traditional child values, namely the “raising children for old-age support” belief and the “continuing the family lineage” belief, exhibit asymmetric states of fulfillment depending on whether a son has been born. For migrant workers who do not yet have male children, both values remain unfulfilled and their subjective importance is typically greater. When job quality improves, the compensatory role of alternative security and identity resources is therefore more likely to emerge. By contrast, for migrant workers who already have male children, both traditional values have, at least in cultural terms, been largely fulfilled, and improvements in job quality represent more of an accumulation of well-being than a substitute for traditional beliefs. Thus, the following hypotheses are proposed:

*Hypothesis 4a:* The association between job quality and weaker endorsement of the “raising children for old-age support” belief is more pronounced among migrant workers without male children than among those with male children.

*Hypothesis 4b:* The association between job quality and weaker endorsement of the “continuing the family lineage” belief is more pronounced among migrant workers without male children than among those with male children.

#### 2.3.5 Gender differences and heterogeneity in resource substitution capacity

In the Chinese context, men and women occupy markedly different structural positions when facing childbearing, and these differences shape whether the resources provided by job quality can be translated into substitutes for traditional child values. Men are typically expected to assume the primary economic responsibility for the family, and their social evaluation is more closely tied to occupational performance, earning capacity, and family provisioning ability. Improvements in job quality are therefore more likely to be associated with alternative security and identity resources, correspondingly reducing dependence on the traditional values of children. By contrast, women’s childbearing-related costs are more direct and concentrated, and the resource gains associated with improved job quality are more easily offset by the anticipated costs of childbearing and caregiving. As a result, the substitution effect may be comparatively weaker. Thus, the following hypothesis is proposed:

*Hypothesis 5:* The negative association between job quality and short-term fertility intentions is more pronounced among male migrant workers than among female migrant workers.

#### 2.3.6 Exploratory heterogeneity analysis of household income level

Families at different income levels may differ in the marginal gains they experience from changes in job quality and in the degree of resource substitution. Because this moderating effect involves the complex interaction of multiple factors, including diminishing marginal utility, opportunity costs of childbearing, and the differential importance of traditional child values across income groups, it is difficult to derive a single, unambiguous prediction from the core theoretical framework. Accordingly, this study does not propose a formal hypothesis regarding the moderating role of household income level but instead examines it as an exploratory heterogeneity analysis.

## 3 Materials and methods

### 3.1 Informed consent statement

Data from the China Family Panel Studies (CFPS) used in this study were submitted to the Peking University Biomedical Ethics Committee for ethical review in accordance with the applicable regulations, and the data collection activity was completed with the approval of the ethical review committee. The approval number was IRB00001052-14010.

In accordance with the ethical principles outlined in the Declaration of Helsinki, all participants gave their informed consent prior to participating in the study. The anonymity and confidentiality of the participants were guaranteed, and participation was entirely voluntary.

### 3.2 Data

This study is based on data from the 2020 China Family Panel Studies (CFPS), a large-scale, multidisciplinary social survey conducted by the Institute of Social Science Survey (ISSS) at Peking University. CFPS is nationally representative and covers all 31 provinces, autonomous regions, and municipalities of China, excluding Taiwan, Hong Kong, and Macau. The survey employs a multi-stage, multi-level random probability sampling design and uses computer-assisted survey techniques to ensure broad national coverage and data quality. The 2020 wave is particularly useful for studying short-term fertility intentions and job quality among Chinese residents. It was conducted following the announcement of the three-child policy, thereby providing an opportunity to examine migrant workers’ fertility intentions in an evolving pro-natalist policy context.

### 3.3 Sample selection

Sample selection followed a multi-step screening procedure based on the definition of migrant workers used by the Chinese National Bureau of Statistics. Starting from the CFPS 2020 adult sample (*N* = 28,530), we first identified migrant workers as individuals with agricultural hukou who had worked in non-agricultural industries for six months or more during the survey year (*N* = 14,816). Because this study focuses on wage-earning migrant workers, whose primary livelihood typically depends on wage income (People’s Daily, 2015), we further restricted the sample to those engaged in paid employment (*N* = 13,475). We then limited the sample to respondents who were currently active in the labor market, excluding those who were unemployed or temporarily out of the labor force (*N* = 11,395).

This study focuses specifically on migrant workers of reproductive age, with special attention to the key role of women in fertility decisions. The CFPS questionnaire on short-term fertility intentions was administered only to respondents aged 17 to 49, yielding *N* = 2,991 migrant workers with valid fertility intention data. In addition, the study is limited to currently married respondents with a living spouse (*N* = 2,973), since childbearing in China remains overwhelmingly concentrated within marriage, and non-marital births are relatively rare (Yu and Xie, 2021). Regarding the upper age limit, research indicates that, compared to younger ages, women aged 45 and above have an 80% higher likelihood of infertility and a natural miscarriage risk as high as 74.7% (Andersen et al., 2000; Jansen, 1984). Following the age limit for women set by de La Rochebrochard and Thonneau (2002) in their study (44 years), the sample in this study includes female respondents aged 44 or younger, and male respondents with female partners aged 44 or younger (*N* = 2,422).

Missing data handling: This study employed listwise deletion to handle missing data. Respondents who chose “refuse to answer,” “not applicable,” or “do not know” for key variables (including fertility intentions, job quality indicators, traditional fertility beliefs, and control variables) were excluded. After these exclusions, the study ultimately obtained a final analytic sample of 2,119 respondents (303 cases excluded due to missing data), including 884 female and 1,235 male participants. To assess potential selection bias, Appendix Table A2 compares the characteristics of the analytic sample with the full eligible sample. The results indicate that the two samples show similar distributions across gender, age, education level, and number of children, suggesting that listwise deletion did not introduce substantial systematic bias.

### 3.4 Dependent variable

The dependent variable is migrant worker’s short-term fertility intention, operationalized through the survey question, “Do you plan to have children in the next two years?” Responses were coded as “Yes” (1) and “No” (0).

This study uses fertility intentions rather than actual fertility behavior (e.g., number of children ever born) as the dependent variable based on the following theoretical and methodological considerations. As a precursor to fertility behavior, fertility intentions have been a core variable in fertility research for nearly 90 years (Westoff and Ryder, 1977). The value of studying fertility intentions rather than fertility behavior lies in three aspects: First, in terms of timeliness, fertility intentions reflect individuals’ current decision-making tendencies and are more sensitive to policy changes and environmental shifts, whereas actual fertility behavior represents historically accumulated outcomes that are less responsive to recent changes such as fluctuations in job quality. Second, in terms of foresight, fertility intentions serve as a leading indicator of future behavior, and studying intentions helps capture the potential direction of future demographic change. Third, in terms of policy relevance, since the goal of policy interventions is to influence future fertility decisions, understanding how intentions are formed has greater policy value than explaining behavior after the fact. Furthermore, this study uses cross-sectional data to analyze the association between job quality and fertility intentions; under this data structure, using historically accumulated fertility behavior as the dependent variable would not accurately capture the association between current working conditions and fertility decision-making.

Regarding the measurement of fertility intentions, compared to indicators reflecting long-term preferences such as “ideal number of children” (Beaujouan and Berghammer, 2019; Hagewen and Morgan, 2005; Westoff and Ryder, 1977), short-term fertility intentions within a 1–3-year time window are widely considered more reliable predictors of actual fertility behavior, as short-term plans better reflect individuals’ immediate assessment of real-life conditions (Billari et al., 2009; Dommermuth et al., 2015; Kuhnt and Trappe, 2016; Schoen et al., 1999). This study adopts the “within the next two years” time frame, which is derived from the design of the CFPS questionnaire and is also consistent with the 1–3-year short-term intention measurement standard commonly used in international fertility research.

It should also be noted that short-term fertility intention, as the dependent variable in this study, primarily reflect individuals’ planful judgments about near-term childbearing under the constraints of current living conditions, family size, and life-course stage, rather than their general preferences for lifetime ideal family size, nor are they equivalent to eventual actual fertility outcomes. Under the cross-sectional design, this measure cannot verify whether intentions are subsequently realized, nor can it distinguish clearly whether the association between job quality and short-term fertility intention reflects more of a timing effect (adjustment in the timing of childbearing) or a quantum effect (a broader orientation toward the number of births). Accordingly, the empirical findings of this study should be understood as associations between job quality and fertility plans within a specific time window, and should not be extrapolated as direct inferences about long-term fertility preferences, completed fertility, or lifetime reproductive trajectories. Further discussion of this limitation is provided in Section 6.

### 3.5 Explanatory variables

#### 3.5.1 Job quality

1 Job Quality Indicator System

This study constructs a composite index to measure migrant worker’s job quality, encompassing six dimensions: wage income (earnings), job stability (job security), work intensity (work hours/intensity), welfare protection (social security coverage), career development prospects (career prospects), and job satisfaction. Specific indicators and their coding schemes are detailed in Table 1.

**Table 1:** Indicators and coding of the job quality index.

Dimension	Sub-dimension	Coding and standardization
Wage income	Hourly wage	Natural logarithm of hourly wage (positively standardized)
Job stability	Labor contract status	No labor contract = 0; short-term contract = 1; long-term contract = 2 (positively standardized)
Work intensity	Weekly working hours	Usual weekly working hours (negatively standardized)
Welfare protection	Pension insurance	Yes = 1; No = 0 (positively standardized)
	Medical insurance	Yes = 1; No = 0 (positively standardized)
	Work-related injury insurance	Yes = 1; No = 0 (positively standardized)
	Unemployment insurance	Yes = 1; No = 0 (positively standardized)
	Maternity insurance	Yes = 1; No = 0 (positively standardized)
Career Development prospects	Promotion opportunities	Yes = 1; No = 0 (positively standardized)
Job satisfaction	Income satisfaction	Satisfaction with income from the current job: very dissatisfied (1) to very satisfied (5) (positively standardized)
	Safety satisfaction	Satisfaction with personal safety at the current job: very dissatisfied (1) to very satisfied (5) (positively standardized)
	Work environment satisfaction	Satisfaction with the work environment of the current job: very dissatisfied (1) to very satisfied (5) (positively standardized)
	Working hours satisfaction	Satisfaction with working hours: very dissatisfied (1) to very satisfied (5) (positively standardized)
	Promotion opportunities satisfaction	Satisfaction with promotion opportunities: very dissatisfied (1) to very satisfied (5); assigned a value of 0 when promotion opportunities are unavailable (positively standardized)

Wage Income: Measured as the natural logarithm of hourly wage.

Job Stability: Evaluated based on the presence of a written labor contract. No contract is coded as 0, short-term contract as 1, and long-term contract as 2.

Work Intensity: Measured as weekly working hours.

Welfare Protection: Assessed by the availability of pension, medical insurance, work-related injury insurance, unemployment insurance, and maternity insurance. Each type of insurance is coded as 1 if provided, otherwise 0. The dimension score is calculated as the mean of these five indicators.

Career Development Prospects: Measured by the presence of promotion opportunities. A promotion opportunity is coded as 1, otherwise as 0.

Job Satisfaction: Includes satisfaction with income, safety, work environment, working hours, and promotion opportunities. Satisfaction is rated on a scale from “very dissatisfied” (1) to “very satisfied” (5). If there is no promotion opportunity, the promotion satisfaction score is coded as 0. The dimension score is calculated as the mean of these five indicators. Coding promotion satisfaction as 0 when no promotion opportunity is available allows the absence of promotion prospects to be incorporated into the index while preserving comparability across respondents.

2 Construction of the Composite Job Quality Index

To reduce bias from subjective weighting, this study uses the entropy weight method, an objective weighting approach based on Shannon entropy, to assign weights at the dimension level. The core principle is that dimensions with greater variation across the sample contain more information and should therefore receive higher weights, whereas dimensions with minimal variation contribute less to distinguishing individuals and should receive lower weights (Shannon, 1948; Xu, 2004; Zeleny, 1982). All sub-indicators were first standardized according to their directional properties. The entropy weight method was then applied to construct the composite job quality index, which was subsequently normalized to range from 0 to 1. The same procedure was applied to the CRITIC-weighted index used in the robustness checks.

Let there be *n* individuals and *m* dimensions (in this study, *m* = 6), with the dimension score matrix $X={\left(x_{\mathrm{ij}}\right)}_{n\times m}$ .

(1) Dimension Transformation and Min-Max Normalization

To ensure comparability and meet the non-negativity requirement of the entropy method, dimensions are first normalized. For positive dimensions (higher values indicate higher job quality):


$$z_{\mathrm{ij}}=\frac{x_{\mathrm{ij}}-\min(x_{j})}{\max(x_{j})-\min(x_{j})}$$

For negative dimensions (higher values indicate lower job quality, such as work intensity):


$$z_{\mathrm{ij}}=\frac{\max(x_{j})-x_{\mathrm{ij}}}{\max(x_{j})-\min(x_{j})}$$

where max(*x_j_*) and min(*x_j_*) represent the maximum and minimum values of dimension *j* in the sample, respectively. If max(*x_j_*) = min(*x_j_*), indicating no variation in that dimension, *z_ij_* is set to 0. This treatment is consistent with standard practices for normalization and comparability in composite indicator construction (Nardo et al., 2008).

(2) Calculating Dimension Proportions

The normalized values are converted to proportions:


$$p_{\mathrm{ij}}=\frac{z_{\mathrm{ij}}}{\sum_{i=1}^{n}z_{\mathrm{ij}}}$$

When $\sum_{i=1}^{n}z_{\mathrm{ij}}=0$ , *p_ij_* is set to 1/*n* to avoid division by zero.

(3) Calculating Information Entropy

$$e_{j}=-k\sum_{i=1}^{n}p_{\mathrm{ij}}\ln p_{\mathrm{ij}},k=\frac{1}{\ln(n)}$$

By convention, when *p_ij_* = 0, $p_{\mathrm{ij}}\ln p_{\mathrm{ij}}=0.$

(4) Calculating Differentiation Coefficients and Determining Weights

A higher entropy value indicates less variation and lower information content. Therefore, the differentiation coefficient is defined as:


$$d_{j}=1-e_{j}$$

The objective weight for dimension *j* is:


$$w_{j}=\frac{d_{j}}{\sum_{j=1}^{m}d_{j}}$$

This weighting logic is consistent with the information-theoretic interpretation that dimensions with greater discriminatory power should receive higher weights (Xu, 2004; Zeleny, 1982).

(5) Calculating the Composite Score

The composite job quality index for individual *i* is:


$$\mathrm{JQ}I_{i}=\sum_{j=1}^{m}w_{i}z_{\mathrm{ij}}$$

The calculation steps and formulas of the entropy weight method described above represent a standard approach widely adopted in composite indicator construction and multi-indicator objective weighting research (Nardo et al., 2008; Shannon, 1948; Xu, 2004; Zeleny, 1982). As a robustness check, the CRITIC method (Diakoulaki et al., 1995) is used as an alternative weighting approach; the corresponding results are reported later in the robustness checks section. Additionally, descriptive statistics and interdimensional correlation analyses for the six dimensions are presented in Appendix Table A1, showing correlation coefficients ranging from 0.136 to 0.541. This suggests that the dimensions are moderately associated without substantial overlap, thereby supporting their use in constructing a composite index.

#### 3.5.2 Endorsement of traditional fertility beliefs

“Raising Children for Old-Age Support” Beliefs: This variable is measured by respondents’ level of agreement with the statement, “Having children is to ensure that someone will assist when I grow old.” Responses range from “strongly disagree” (1) to “strongly agree” (5), with higher values indicating stronger agreement with this belief.

“Continuing the Family Lineage” Beliefs: Similarly, this variable is assessed based on respondents’ agreement with the statement, “Having children is to continue the family bloodline.” The response scale follows the same format, from “strongly disagree” (1) to “strongly agree” (5), with higher values reflecting a stronger endorsement of this belief.

It should be noted that both belief variables are measured using single items. This measurement approach reflects the design of the CFPS questionnaire; as a large-scale national survey, it is constrained by questionnaire length and respondent burden, which precluded the inclusion of multi-item scales. Although single-item measures have limitations in capturing the multidimensionality of complex cultural constructs, the items used in this study directly assess respondents’ endorsement of these two core fertility beliefs and can meaningfully distinguish individuals in their endorsement of them. Furthermore, these two items correspond closely to the core concepts in the theoretical framework: the “raising children for old-age support” item directly measures the perceived instrumental value of children as resources for old-age security, whereas the “continuing the family lineage” item captures the endorsement of family continuation as a cultural responsibility. Further limitations of the single-item measurement approach are discussed in the Limitations section.

### 3.6 Control variables

Control variables are grouped into two categories: individual characteristics and family characteristics. Individual characteristics include gender (male = 1, female = 0), age, age squared, education level (ranging from illiterate/semi-illiterate = 1 to PhD = 8), education squared, and self-rated health (from 1 = very healthy to 5 = unhealthy). To reduce potential multicollinearity arising from the inclusion of squared terms, age and education were mean-centered before being entered into the regression models (Aiken et al., 1991). Family characteristics include parity, defined as the number of currently living children at the time of the survey, logged household per capita net income over the past 12 months, spouse’s age, spouse’s education level coded using the same scale as the respondent’s education, and spouse co-residence, coded as 1 if the spouse lives with the respondent and 0 otherwise.

It should be noted that some of the above control variables are also used as grouping variables in subsequent analyses. Specifically, parity is used for the parity-stratified core analysis to examine whether the association between job quality and short-term fertility intentions varies by current family size. In addition, gender is also used as a grouping variable in the heterogeneity analysis. For the income-based heterogeneity analysis, household income level is categorized into low-income (bottom 25th percentile), middle-income (middle 50th percentile), and high-income (top 25th percentile) groups based on the national household per capita income quartile distribution provided in the CFPS data. In the above analyses, subgroup regressions are used, with the sample divided into different subgroups based on these variables to examine whether the relevant associations vary across groups.

Table 2 provides the specific definitions and values for these variables.

**Table 2:** Definitions of variables.

Variable name	Variable symbol	Definition and coding
Dependent variable		
Short-term fertility intention	Fertility intention	Whether the respondent intends to have children within the next two years: Yes = 1; No = 0
Explanatory variables		
Job quality index	Job quality index	Entropy-weighted composite index of job quality based on six dimensions; normalized to range from 0 to 1
CRITIC-weighted job quality index	CRITIC-weighted job quality index	Alternative composite index constructed using the CRITIC weighting method for robustness checks; normalized to range from 0 to 1
“Raising Children for Old-Age Support” belief	Old-age support belief	Respondent’s level of agreement with the statement, “Having children is to ensure that someone will assist when I grow old”: strongly disagree (1) to strongly agree (5)
“Continuing the Family Lineage” belief	Family lineage belief	Respondent’s level of agreement with the statement, “Having children is to continue the family bloodline”: strongly disagree (1) to strongly agree (5)
Control variables		
Gender	Gender	Respondent’s gender: Male = 1; Female = 0
Age	Age	Respondent’s age in years; continuous variable
Age squared	Age squared	Square of the respondent’s age; continuous variable
Education level	Education	Highest educational level completed by the respondent (illiterate/semi-illiterate = 1; primary school = 2; junior high school = 3; senior high school/technical school/vocational school = 4; junior college = 5; bachelor’s degree = 6; master’s degree = 7; PhD = 8)
Education squared	Education squared	Square of the respondent’s educational level; continuous variable
Self-rated health	Self-rated health	Respondent’s self-rated health: very healthy = 1 to unhealthy = 5
Spouse’s age	Spouse age	Age of the respondent’s spouse in years; continuous variable
Spouse’s education level	Spouse education	Educational level of the respondent’s spouse, coded using the same scale as the respondent’s education
Spouse co-residence	Spouse co-residence	Whether the spouse lives with the respondent: Yes = 1; No = 0
Parity	Parity	Number of currently living children at the time of the survey; continuous variable
Logged household per capita net income	ln household income	Natural logarithm of household per capita net income over the past 12 months (yuan); continuous variable

Unless otherwise stated, job quality index refers to the entropy-weighted Job Quality Index used in the baseline analysis. CRITIC-weighted job quality index is used only for robustness checks.

### 3.7 Analytical strategy

A two-level mixed-effects logistic regression model is employed to assess the association between job quality and migrant worker’s short-term fertility intention, given the binary nature of the dependent variable (Goldstein, 2011; Hox et al., 2017; Novelli et al., 2021). This model is well suited to the present analysis because substantial economic, cultural, and policy differences across provinces in China may be associated with fertility intention. The first level includes individual-level variables, encompassing both personal and family characteristics, whereas the second level accounts for provincial heterogeneity through random intercepts at the provincial level. This hierarchical approach helps account for unobserved provincial heterogeneity and improves the precision of the estimates. Data analysis was conducted using Stata 17.0.

## 4 Results

### 4.1 Descriptive results

This study focuses on migrant workers of reproductive age. Respondents range in age from 17 to 44 years, with a mean age of 34.13, indicating that the sample remains within the age range relevant to childbearing. However, 83.9% of migrant workers reported no plans to have children within the next two years.

Regarding job quality, the mean values of the entropy-weighted and CRITIC-weighted job quality indices are 0.46 and 0.49, respectively, with standard deviations of 0.18 and 0.20, indicating only slight differences between the two weighting methods. In terms of family structure, 45.8% of migrant workers have one child, 43.3% have two children, and 10.5% have three or more children, whereas only 0.4% remain childless. This parity distribution indicates that most migrant workers in the sample have already had a first child, and their short-term fertility intention primarily concerns whether to continue childbearing rather than whether to have a first child. The extremely small childless group (*n* = 9), and the analytical constraints this creates, are discussed further below.

Table 3 reports the descriptive statistics for the main variables.

**Table 3:** Descriptive statistics.

Variables	Mean	Std. Dev.	Min	Max	Obs.
Fertility intention	0.16	0.37	0	1	2,119
Job quality index	0.46	0.18	0	1	2,119
CRITIC-weighted job quality index	0.49	0.20	0	1	2,119
Old-age support belief	2.84	0.65	1	5	2,119
Family lineage belief	2.73	0.67	1	5	2,119
Gender	0.58	0.49	0	1	2,119
Age	34.13	5.70	17	44	2,119
Age squared	1,197.60	400.85	289	2,401	2,119
Education	3.69	1.24	1	7	2,119
Education squared	15.11	9.86	1	49	2,119
Self-rated health	2.58	1.00	1	5	2,119
Spouse age	33.98	5.84	19	55	2,119
Spouse education	3.59	1.28	1	8	2,119
Spouse co-residence	0.91	0.28	0	1	2,119
Parity	1.66	0.74	0	9	2,119
ln household income	10.05	0.78	7.13	14.51	2,119

Data are from the China Family Panel Studies (CFPS) (2020); authors’ calculations.

### 4.2 Baseline results: the overall association between job quality and short-term fertility intention

Table 4 presents the regression results examining the association between the job quality index and migrant workers’ short-term fertility intention. The results show that job quality is significantly negatively associated with the likelihood of intending to have children within the next two years (*B* = −0.861, *p* < 0.1, OR = 0.423), providing preliminary support for Hypothesis 1. In addition, the province-level random intercept is significant at the 1% level, suggesting that provincial differences are significantly associated with the dependent variable. The LR test (chibar2(01) = 9.180, *p* = 0.001) further indicates that the mixed-effects model is more appropriate than a standard logistic regression model.

**Table 4:** Association between job quality and short-term fertility intention: two-level mixed-effects logistic regression.

Variable	Coef.	OR	Std. Err.	*p*-value	90% CI
Job quality index	−0.861*	0.423*	0.508	0.090	[−1.697, −0.026]
Gender	0.577***	1.781***	0.160	0.000	[0.314, 0.840]
Age	0.229	1.257	0.154	0.137	[−0.024, 0.482]
Age squared	−0.005**	0.995**	0.002	0.034	[−0.009, −0.001]
Education	−0.585*	0.557*	0.341	0.086	[−1.147, −0.024]
Education squared	0.080**	1.083**	0.039	0.042	[0.015, 0.145]
Self-rated health	0.070	1.072	0.072	0.334	[−0.049, 0.188]
Spouse age	−0.040	0.961	0.026	0.129	[−0.084, 0.003]
Spouse education	0.110	1.117	0.074	0.136	[−0.011, 0.231]
Spouse co-residence	0.226	1.253	0.286	0.430	[−0.245, 0.696]
Parity	−1.790***	0.167***	0.175	0.000	[−2.077, −1.502]
ln household income	0.217*	1.242*	0.112	0.053	[0.032, 0.401]
Constant	−2.057	0.128	2.793	0.461	[−6.650, 2.536]
Random-intercept variance (province)	0.150***	—	0.097	0.001	[0.052, 0.436]
LR test (chibar^2^, *p*)	9.180, *p* = 0.001				
Obs.	2,119				
Groups: Provinces	28				
Wald chi2	280.120				
Log likelihood	−693.946				

^a^Data are from the China Family Panel Studies (CFPS) (2020); authors’ calculations.

^b^***p < 0.01, **p < 0.05, *p < 0.10.

^c^Std. Err., *p*-values, and 90% confidence intervals are reported for coefficients. Odds ratios (OR) are obtained by exponentiating the coefficients (OR = exp[Coef.]).

Control variable analysis reveals that male migrant workers are more likely to plan to have children than females within the next two years. Short-term fertility intention is positively associated with education squared and household per capita net income, but negatively associated with age squared, education, and parity. No significant associations were found for other control variables and fertility intentions.

### 4.3 Parity-stratified Core analysis

As discussed in Section 2.2.4, short-term fertility intention is embedded in the specific context of family size and life-course stage, and the association between job quality and fertility intention may therefore differ across parity groups. This section stratifies the sample by parity and estimates the main model separately for each group to test Hypothesis 2, namely whether the negative association is more pronounced among one-child families facing the active decision of whether to have a second child.

Because the childless group is very small (*n* = 9), regression estimates cannot be obtained reliably; therefore, intentions to have a first child within the next two years cannot be examined. This limitation means that the present study cannot draw conclusions about the association between job quality and first-birth decisions, and future research should revisit this question using larger childless samples.

Table 5 presents regression results for three parity groups: one child (*n* = 970), two children (*n* = 918), and three or more children (*n* = 222). The results show that the job quality index is significantly negatively associated with the fertility intention of migrant workers with one child to have a second child within the next 2 years (*B* = −1.382, *p* = 0.017). By contrast, the job quality index is not significantly associated with fertility intention in either the two-child group or the three-or-more-children group. This finding is consistent with Hypothesis 2 and has important implications for interpreting the full-sample results reported in Section 4.2: the overall negative association observed in the full sample is not evenly distributed across parity groups but is primarily concentrated among one-child families facing the decision of whether to have a second child.

**Table 5:** Association between job quality and short-term fertility intention by parity: two-level mixed-effects logistic regression.

Variable	One child	Two children	Three or more children
Job quality index	−1.382**	1.911	2.705
	(0.577)	(1.443)	(3.450)
	[−2.363, −0.461]	[−0.490, 4.248]	[−2.945, 8.429]
Control variables	Yes	Yes	Yes
Constant	−9.636***	10.571	−81.217
	(3.447)	(7.434)	(59.733)
Random-intercept variance (province)	0.189***	0.002	0.000
	(0.120)	(0.295)	(0.000)
LR test (chibar^2^, *p*)	9.350, *p* = 0.001	0.000, *p* = 0.498	0.000, *p* = NA
Obs.	970	918	222

^a^All models are estimated using two-level mixed-effects logistic regression.

^b^Data are from the China Family Panel Studies (CFPS) (2020); authors’ calculations.

^c^Coefficients are reported, standard errors are in parentheses, and 90% confidence intervals are in brackets.

^d^***p < 0.01, **p < 0.05, *p < 0.10.

^e^The control variables are identical to those in Table 4.

^f^The childless group, due to its small size (only nine), could not yield reliable regression estimates.

### 4.4 Robustness checks

#### 4.4.1 Control function approach

Although the baseline model controls for a range of factors related to migrant workers’ fertility intention, residual bias may still arise from unobserved variables. Because women may sort into lower-quality jobs in order to balance motherhood and work roles (Piasna and Plagnol, 2018), fertility intention may in turn be associated with job quality. To address this potential endogeneity, the study adopts a control function approach. Specifically, the average job quality index of other migrant workers within the same county is used as an instrumental variable. This indicator reflects the local employment environment and is highly correlated with individual job quality, while being unlikely to be directly associated with fertility intention. As shown in Table 6, the residual term is significant at the 10% level, suggesting the presence of endogeneity in the baseline model. After correcting for this issue, the negative association between the job quality index and migrant workers’ short-term fertility intention remains significant.

**Table 6:** Robustness checks: control function, CRITIC method, and age restriction for women under 40.

Variable	Control function approach	CRITIC-weighted job quality index	Age restriction for women (under 40 years)
Job quality index	−5.782*	−1.087**	−0.910*
	(2.988)	(0.516)	(0.543)
	[−10.715, −0.846]	[−1.931, −0.231]	[−1.823, −0.112]
Residual (endogeneity correction variable)	5.131*	—	—
	(2.994)	—	—
	[0.148, 10.038]	—	—
Control variables	Yes	Yes	Yes
Constant	−3.846	−1.807	−1.097
	(3.487)	(2.771)	(3.490)
Random-intercept variance (province)	0.168***	0.152***	0.192***
	(0.110)	(0.098)	(0.116)
LR test (chibar^2^, *p*)	9.980, p = 0.001	9.370, p = 0.001	11.600, *p* < 0.001
Obs.	1,979	2,119	1,633

^a^All models are estimated using two-level mixed-effects logistic regression.

^b^Data are from the China Family Panel Studies (CFPS) (2020); authors’ calculations.

^c^Coefficients are reported, standard errors are in parentheses, and 90% confidence intervals are in brackets.

^d^***p < 0.01, **p < 0.05, *p < 0.10.

^e^The control variables are identical to those in Table 4.

#### 4.4.2 Modification of job quality measurement

The baseline model uses the entropy-weighted job quality index. To assess robustness, we re-estimate the model using the CRITIC-weighted job quality index. As reported in Table 6, the CRITIC-weighted Job Quality Index remains significantly negatively associated with migrant workers’ short-term fertility intention, further confirming the robustness of the baseline results.

#### 4.4.3 Narrowing the age range of women of reproductive age

Given the marked decline in fertility potential among women aged 40 and above (Qiao et al., 2014), we conduct an additional robustness check by restricting the sample to women under age 40. This restriction includes all female respondents under 40 as well as female partners of male respondents under the same age threshold. Table 6 shows that the job quality index remains significantly negatively associated with fertility intention within the next two years, further supporting the robustness of the main findings.

### 4.5 Mechanism analysis: associational pathways through traditional fertility beliefs

#### 4.5.1 Job quality and the “Raising Children for Old-Age Support” belief

According to the regression results in Table 7, in the full sample the job quality index is not significantly associated with the “raising children for old-age support” belief. However, among migrant workers without male children, the job quality index is significantly negatively associated with endorsement of this belief (*B* = −1.539, *p* = 0.009). Among migrant workers who already have male children, the association is not significant. Thus, Hypothesis 3a is not supported in the full sample, whereas the pattern predicted by Hypothesis 4a, namely that the association is more pronounced among those without male children, is consistent with the data.

**Table 7:** Association between job quality and endorsement of traditional fertility beliefs: mechanism analysis.

Variable	Total sample	Without male children	With male children
“Raising Children for Old-Age Support” Belief			
Job quality index	−0.260	−1.539***	0.265
	(0.324)	(0.592)	(0.388)
	[−0.792, 0.273]	[−2.512, −0.565]	[−0.374, 0.903]
Control variables	Yes	Yes	Yes
Obs.	2,119	636	1,483
***“*** Continuing the Family Lineage” Belief			
Job quality index	−0.687**	−1.036*	−0.599
	(0.322)	(0.583)	(0.389)
	[−1.241, −0.184]	[−1.995, −0.077]	[−1.280, 0.002]
Control variables	Yes	Yes	Yes
Obs.	2,119	636	1,483

^a^All models are estimated using two-level mixed-effects ordered logistic regression.

^b^Data are from the China Family Panel Studies (CFPS) (2020); authors’ calculations.

^c^Coefficients are reported, standard errors are in parentheses, and 90% confidence intervals are in brackets.

^d^***p < 0.01, **p < 0.05, *p < 0.10.

^e^The control variables are identical to those in Table 4.

#### 4.5.2 Job quality and the “Continuing the Family Lineage” belief

The results in Table 7 show that the job quality index is significantly negatively associated with migrant workers’ endorsement of the “continuing the family lineage” belief at the 5% level (*B* = −0.687, *p* = 0.033), providing support for Hypothesis 3b. The subgroup analysis further indicates that this association is significant at the 10% level among migrant workers without male children (*B* = −1.036, *p* = 0.076), whereas it is not significant among those who already have male children. This pattern is consistent with the predicted direction of Hypothesis 4b.

Taken together, the two mechanism pathways receive uneven empirical support. The economic security pathway (H3a) is not supported in the full sample but is significant in the subgroup without male children, whereas the social recognition pathway (H3b) is supported in the full sample. The heterogeneity patterns for both pathways by the presence or absence of male children (H4a and H4b) are consistent with theoretical expectations. Overall, these findings provide partial empirical support for traditional fertility beliefs as potential psychological mechanisms underlying the association between job quality and fertility intention, but they do not fully validate the proposed mechanism framework. This limitation is discussed further in Section 5.

### 4.6 Further heterogeneity analysis

#### 4.6.1 Gender heterogeneity

The subgroup regression results in Table 8 indicate that gender significantly moderates the association between job quality and fertility intention. Among male migrant workers, the job quality index is significantly negatively associated with the intention to have children within the next two years (*B* = −1.337, *p* = 0.039). Among female migrant workers, the job quality index is not significantly associated with fertility intention within the same time frame. This finding is consistent with Hypothesis 5 and suggests that the resources provided by job quality may substitute for traditional child values more strongly among men.

**Table 8:** Association between job quality and short-term fertility intention: heterogeneity analysis.

Variable	Gender		Household income level		
	Male	Female	Low-income	Middle-income	High-income
Job quality index	−1.337**	0.152	−0.828	−1.060*	−0.512
	(0.647)	(0.833)	(2.496)	(0.643)	(1.037)
	[−2.480, −0.343]	[−1.211, 1.524]	[−4.933, 3.279]	[−2.184, −0.062]	[−2.227, 1.184]
Control variables	Yes	Yes	Yes	Yes	Yes
Obs.	1,235	884	265	1,344	510

^a^All models are estimated using two-level mixed-effects logistic regression.

^b^Data are from the China Family Panel Studies (CFPS) (2020); authors’ calculations.

^c^Coefficients are reported, standard errors are in parentheses, and 90% confidence intervals are in brackets.

^d^***p < 0.01, **p < 0.05, *p < 0.10.

^e^The control variables are identical to those in Table 4.

^f^The migrant workers are categorized into three income groups based on the national household per capita income quartile distribution in the CFPS data: low-income (bottom 25th percentile), middle-income (middle 50%), and high-income (top 25th percentile).

#### 4.6.2 Exploratory analysis of household income level

The results in Table 8 show that the job quality index is not significantly associated with fertility intention among migrant workers from either low-income or high-income households. By contrast, a significant negative association is observed among those from middle-income households, for whom the job quality index significantly reduces fertility intention within the next two years (*B* = −1.060, *p* = 0.099). As an exploratory finding, this pattern is consistent with the direction suggested by COR theory and the law of diminishing marginal utility, implying that middle-income families may occupy the range in which the marginal utility of resources is most sensitive. However, given the exploratory nature of this analysis, the result should be interpreted with caution.

## 5 Discussion

Using 2020 CFPS data, this study examined the association between job quality and short-term fertility intentions among Chinese migrant workers and explored the role of traditional fertility beliefs as potential psychological mechanisms underlying this association. The discussion below is organized around the main findings.

### 5.1 The overall association between job quality and short-term fertility intentions and its scope conditions

The full-sample analysis shows that job quality is significantly negatively associated with migrant workers’ short-term fertility intentions within the next 2 years (Hypothesis 1). This result remains robust when the CRITIC weighting method is used instead of the entropy method, when a control function approach is applied to address potential endogeneity, and when the age range for women is narrowed. Moreover, the significant province-level random intercept indicates that cross-provincial variation in economic, cultural, and policy conditions is also associated with variation in fertility intention.

However, the parity-stratified core analysis further reveals the scope of applicability of this association: the negative association between job quality and short-term fertility intentions is primarily concentrated among one-child families facing the decision of “whether to have a second child” (Hypothesis 2), whereas no significant association is observed among families with two children or three or more children. This result indicates that the above association does not hold equally across all family size contexts, and is also consistent with the theoretical analysis in Section 2.2.4: given that the first child carries strong emotional and social normative value, the alternative resources provided by job quality are more likely to matter at the active decision point at which one-child families face the question of whether to continue childbearing. This finding suggests that, in the context of the three-child policy, understanding changes in second-birth intentions among one-child families may require closer attention to how employment conditions are associated with their short-term fertility plans.

Furthermore, as noted in Section 3.4, the dependent variable of this study, short-term fertility intentions within the next 2 years, captures planful judgments within a specific time window and is not equivalent to long-term fertility preferences or actual fertility outcomes. Accordingly, the above findings should not be directly extrapolated as inferences about lifetime reproductive trajectories. At the same time, under a cross-sectional design, this study cannot clearly distinguish whether the observed association more closely reflects an adjustment in the timing of childbearing (a timing effect) or a change in broader fertility quantity orientation (a quantum effect). Future research should employ longitudinal data to further examine the dynamic relationship between changes in job quality and the realization of fertility intentions.

The above findings form a noteworthy contrast with the conclusions of Hanappi et al. (2016), who found that among Swiss couples, improved job quality was associated with stronger fertility intentions. This difference can be understood in terms of differences in the structure of child values across cultural contexts. Compared to European countries such as Switzerland, traditional functional child values remain to some extent embedded in the belief systems of Chinese migrant workers; accordingly, the resource substitution associated with improved job quality may therefore be linked to short-term fertility intentions in a different direction. This cross-cultural difference also echoes Nauck’s (2005, 2007) argument that the composition of child values undergoes systematic change across stages of socioeconomic development, and that the direction and strength of its association with fertility decisions may also vary by cultural context.

### 5.2 Traditional fertility beliefs as mechanisms: partial evidence

This study proposed two associational pathways, the economic security pathway and the social recognition pathway, to explain how job quality may be linked to fertility intention through the reshaping of traditional child value assessments. The empirical results provide differing degrees of support for these two pathways.

The social recognition pathway receives relatively consistent support: the job quality index is significantly negatively associated with migrant workers’ endorsement of the “continuing the family lineage” belief in the full sample (Hypothesis 3b). This result is consistent with the theoretical expectation of this study, namely that the expansion of identity resources and social recognition pathways accompanying high-quality employment may be linked to a weakened psychological need to obtain social recognition through bearing sons.

The evidence for the economic security pathway is more limited: the association between job quality and the “raising children for old-age support” belief is not significant in the full sample (Hypothesis 3a is not supported). This result suggests that, for migrant workers as a whole, the economic security resources provided by job quality may not yet be sufficient to significantly substitute for the functional role of children in old-age security at the aggregate level. This may reflect the fact that social insurance coverage among Chinese migrant workers remains relatively limited and that institutionalized old-age security systems are not yet fully developed. Under such conditions, even when job quality improves, individuals’ dependence on the old-age security function of children may be difficult to fully substitute.

However, the heterogeneity analysis along the dimension of presence or absence of male children reveals a more nuanced pattern. For migrant workers without male children, the association between job quality and weakened endorsement of both the “raising children for old-age support” and “continuing the family lineage” beliefs is significant (Hypotheses 4a, 4b); for migrant workers who already have male children, neither association is significant. This pattern is consistent with the theoretical expectation that when traditional cultural responsibilities have, to some extent, already been fulfilled, the substitution effect associated with improved job quality becomes weaker.

It is worth noting that, among migrant workers who already have male children, the lack of a significant association between improved job quality and weakened endorsement of the “raising children for old-age support” belief also suggests that once a family has a son, the family-based old-age support model retains considerable persistence in this group. This pattern resonates to some extent with Inglehart and Baker’s (2000) argument that cultural heritage continues to exert influence despite modernization. Therefore, improvements in job quality may be associated to some extent with the weakening of traditional fertility beliefs, but do not fully substitute for these beliefs, particularly among migrant worker families that already have male children.

Overall, the above results provide partial empirical support for traditional fertility beliefs as potential psychological mechanisms underlying the association between job quality and fertility intentions. The two mechanism pathways receive uneven support: the evidence for the social recognition pathway is relatively consistent, while the evidence for the economic security pathway is more prominent in specific subgroups. At the same time, it should be acknowledged that the theoretical arguments of this study concerning resource substitution, value reassessment, and changes in sources of social recognition are considerably broader than what the current data can directly verify. Both belief variables are measured with single items, which limits their capacity to fully capture the multidimensionality of these complex cultural constructs. Future research should consider developing multi-item scales to more comprehensively measure different facets of traditional fertility beliefs, thereby enabling a more thorough test of the proposed mechanism framework.

### 5.3 Interpretation of the findings from a value of children perspective

From a Value of Children (VOC) perspective, the findings of this study suggest that the association between job quality and short-term fertility intentions is not merely a direct manifestation of changes in employment conditions. Rather, it is more likely to accompany adjustments in individuals’ subjective assessments of the functional values of children. Furthermore, among Chinese migrant workers, traditional functional child values have not entirely disappeared from their belief systems, but their relative importance may shift as employment resources improve. This finding is consistent with the classic proposition of Hoffman and Hoffman (1973) that children carry multiple values, and also aligns with Nauck’s (2005, 2007) theoretical expectation that the composition of child values is reconfigured in response to institutional environments and developmental stages.

This study further introduces the resource substitution perspective of COR theory within the VOC framework to show how the economic security, social recognition, and identity-related resources associated with job quality may, to some extent, substitute for the functions traditionally fulfilled by children. This resource substitution perspective also provides an explanation closer to individuals’ lived circumstances for understanding the macro-level arguments within VOC theory regarding the transformation of child values.

At the same time, this study focuses on Chinese migrant workers, a particular group situated at the intersection of tradition and modernity. The pattern observed in this group, namely that the weakening of traditional functional child values is linked to the substitution effect accompanying improved employment resources, while these beliefs remain relatively persistent among those who already have male children, suggests that the transformation of child values does not proceed in a linearly but may unfold at different paces across different dimensions, constrained by the status of cultural responsibility fulfillment. This finding provides an empirical reference for the applicability and boundary conditions of VOC theory in non-Western, transitional society contexts.

However, the boundaries between this study and the broader VOC literature should also be noted. VOC theory typically addresses the full spectrum of child values (including emotional value, stimulation and fun value, etc.), whereas this study, due to the constraints of the CFPS questionnaire design, examines only two categories of functional values (economic security and social recognition) and does not address the emotional value dimension of children. This means that the findings of this study reflect only the association between the functional value dimension of the child value spectrum and employment conditions, and should not be regarded as a complete account of the overall transformation of child value structures. Future research may incorporate more comprehensive measures of child values to examine whether job quality exhibits differentiated associational patterns across different dimensions of child values.

### 5.4 Supplementary findings from heterogeneity analyses

The gender heterogeneity analysis indicates that the negative association between job quality and short-term fertility intentions is significant among male migrant workers, while no significant association is found among female migrant workers (Hypothesis 5). Within the theoretical framework of this study, this gender difference can be understood in terms of differences in the capacity of job-quality-related resources to substitute for traditional child values. Men are typically expected to assume the primary economic responsibility for the family, and their social evaluation is more closely tied to occupational performance; accordingly, improvements in job quality are more likely to be associated with alternative security resources and identity resources. By contrast, women’s childbearing-related costs are more direct and concentrated, and the resource gains from improved job quality are more easily offset by the anticipated costs of childbearing and caregiving. This result is also consistent with the direction of Novelli et al.’s (2021) finding that work conditions may be more closely associated with fertility intentions among men than among women. At the same time, this also suggests that understanding women’s fertility intentions may require going beyond job quality itself, with further attention to the joint influence of social support, workplace policies, and gender role norms (Han, 2023; Han et al., 2024; Testa et al., 2011; Yoon, 2016; Yu and Kuo, 2017).

The exploratory analysis of household income level shows that the job quality index is significantly negatively associated with short-term fertility intentions among middle-income family migrant workers, whereas no significant association is found among those from low-income or high-income families. This exploratory finding is consistent with the basic logic of COR theory and the law of diminishing marginal utility, namely that groups at different income levels may differ in their sensitivity to the marginal resource gains brought about by improved job quality. However, given the exploratory nature of this analysis and the complex interaction of multiple factors involved in the theoretical reasoning, the interpretation of this result should remain cautious and awaits further examination in future research.

## 6 Conclusion, implications and limitations

### 6.1 Conclusion

Using data from the 2020 China Family Panel Studies (CFPS), this study developed an analytical framework centered on Value of Children theory and informed by Conservation of Resources theory and social identity theory to examine the association between job quality and short-term fertility intentions among Chinese migrant workers, and to explore the potential role of traditional fertility beliefs in this association. The main findings can be summarized as follows.

First, job quality is significantly negatively associated with migrant workers’ short-term fertility intention within the next 2 years, and this result remains consistent across multiple robustness checks. Second, the parity-stratified analysis indicates that this association is primarily concentrated among one-child families and is not significant among families with two or more children, suggesting that normative family size constitutes an important scope condition. Third, at the mechanism level, the social recognition pathway receives relatively consistent support, whereas support for the economic security pathway is more prominent in specific subgroups. Specifically, a significant association is found between job quality and weakened endorsement of the “continuing the family lineage” belief in the full sample, whereas the association between job quality and weakened endorsement of the “raising children for old-age support” belief is significant only among migrant workers without male children. Fourth, the negative association between job quality and short-term fertility intentions is primarily observed among male migrant workers, whereas no significant association is found among female migrant workers.

Overall, the findings suggest that the association between job quality and migrant workers’ short-term fertility intention is not exhausted by changes in employment conditions alone, but may also be accompanied by adjustments in the subjective assessment of traditional child values. However, this association is conditional and context-dependent: it is primarily concentrated in specific parity stages, gender groups, and cultural circumstances, and the current evidence regarding mechanism pathways remains partial.

### 6.2 Implications

Based on the above findings, this study may offer several points of reference for policy discussions. Because this study employs a cross-sectional design, the following implications should be understood as preliminary, directional suggestions that await further empirical verification.

First, the finding that the negative association between job quality and fertility intentions is primarily concentrated among one-child families suggests that the effective implementation of the three-child policy may require closer attention to the trade-off between employment conditions and second-birth decisions among one-child families. For one-child families, providing targeted economic support to reduce the burden of child-rearing, while simultaneously fostering a supportive fertility environment at the socio-cultural level, may help alleviate the tension between improved employment conditions and declining fertility intentions.

Second, the gender differences in the association between job quality and fertility intentions suggest that improving women’s fertility intentions may depend not only on improvements in employment conditions but also on concurrent efforts to reduce the asymmetric burden women bear in childbearing, caregiving, and housework, to promote a more balanced division of labor within households, and to strengthen gender-supportive workplace policies.

Third, the exploratory finding regarding middle-income families suggests that this group may be more sensitive to resource changes in their fertility decisions. Policy measures such as fertility subsidies, reductions in educational expenses, and career development support may offer useful policy directions for alleviating fertility-related pressures among middle-income families, although this inference still requires further empirical verification.

### 6.3 Limitations

This study has several limitations that should be taken into account when interpreting the findings.

First, there are limitations related to identification strategy and causal inference. This study uses cross-sectional data, and therefore all results can only be interpreted as associations rather than causal relationships. The direction of the relationship between job quality and fertility intentions is fundamentally uncertain: better job quality may be associated with lower fertility intentions, but conversely, individuals with childbearing plans may also proactively choose more flexible but lower-quality jobs, or adjust their labor market participation strategies, based on anticipated future allocations of time and energy (Piasna and Plagnol, 2018). Furthermore, job quality and fertility intentions may both be influenced by unobserved factors, including personal values, career orientations, health status, family background, and spousal characteristics. Although this study adopted a control function approach to address potential endogeneity and controlled for a range of individual and family characteristics, these methods can only mitigate rather than fully resolve the endogeneity problems inherent in cross-sectional data. Therefore, the findings should be understood as evidence of association between job quality and short-term fertility intentions, rather than as support for strong causal claims. Future research should employ panel data or natural experimental designs to identify more reliably the directional relationship between changes in job quality and changes in fertility intentions.

Second, there are limitations in the measurement of the dependent variable and in the scope of result interpretation. As discussed in Section 3.4, the dependent variable in this study is short-term fertility intention within the next 2 years, and this measure captures planful judgments within a specific time window. It is not equivalent to intended family size, ideal family size, or eventual realized fertility. At the same time, under a cross-sectional design, this study cannot clearly distinguish whether the observed association more closely reflects an adjustment in the timing of childbearing or a change in fertility quantity orientation. Accordingly, the findings should be delimited as associations between job quality and fertility plans within a specific time window and should not be extrapolated to long-term fertility preferences or lifetime reproductive trajectories. Future research should use longitudinal data to track the realization of fertility intentions, in order to assess more precisely the dynamic relationship between changes in job quality and actual fertility behavior.

Third, there are limitations in the measurement of the mechanism variables. The two variables used to measure traditional fertility beliefs, namely the “raising children for old-age support” belief and the “continuing the family lineage” belief, were both assessed with single items. This measurement approach reflects the constraints of the CFPS questionnaire in terms of survey length and respondent burden. Single-item measures may not fully capture the multidimensionality of these complex cultural constructs and may therefore underestimate the actual role of traditional fertility beliefs in the association between job quality and fertility intentions. In addition, as discussed in Section 5.2, the theoretical arguments advanced in this study remain broader than what the current data can directly verify; accordingly, the discussion of mechanism pathways should be understood as theoretical interpretations supported by partial evidence. Future research should consider developing multi-item scales to measure more comprehensively different facets and levels of intensity in traditional fertility beliefs, thereby enabling a more thorough test of the proposed mechanism framework.

Fourth, there are limitations related to the sample and subgroup analyses. The sample is restricted to migrant workers of childbearing age, and some key subgroups are relatively small, which may constrain the generalizability of the findings. As noted in Section 4.3, the childless group is extremely small (*n* = 9) and cannot yield reliable regression estimates, which means that the theoretical inference regarding parity-based scope conditions has not been fully tested. Although the CFPS data are nationally representative, lending a degree of reliability to the conclusions of this study, future research should further examine the present findings using larger samples and more balanced subgroup distributions. In addition, future research may explore more fully the differentiated associations between specific dimensions of job quality, such as promotion opportunities, income satisfaction, and welfare protection, and fertility intentions, in order to achieve a more nuanced understanding of the complex relationship between employment conditions and fertility decisions.

## Data Availability

Publicly available datasets were analyzed in this study. This data can be found at: the data supporting the findings of this study are available from the China Family Panel Studies (CFPS). Access to the CFPS dataset requires registration and approval, which can be requested at: https://www.isss.pku.edu.cn/cfps/.
