# Supplementary material for: Job quality and fertility intentions among Chinese migrant workers: the role of traditional fertility beliefs
Source: Front Psychol. 2026 Jun 17;17:1739790. doi: 10.3389/fpsyg.2026.1739790 (PMC13320662; doi:10.3389/fpsyg.2026.1739790)
Supplement: Supplementary file 1 [file Data_Sheet_1.docx]

**Table S1.** Association between job quality and short-term fertility intention: one child

| **Variables** | **Coef.** | **Std. Err.** | **p-value** | **90% CI** |
| --- | --- | --- | --- | --- |
| Job quality index | −1.382** | 0.577 | 0.017 | [−2.363, −0.461] |
| Gender | 0.606*** | 0.178 | 0.001 | [0.313, 0.898] |
| Age | 0.516** | 0.204 | 0.011 | [0.181, 0.851] |
| Age squared | −0.009*** | 0.003 | 0.003 | [−0.015, −0.004] |
| Education | 0.042 | 0.098 | 0.666 | [−0.119, 0.204] |
| Education squared | 0.036 | 0.049 | 0.459 | [-0.044, 0.116] |
| Self-rated health | 0.019 | 0.083 | 0.822 | [−0.119, 0.156] |
| Spouse age | −0.060** | 0.030 | 0.047 | [−0.110, −0.010] |
| Spouse education | 0.148* | 0.082 | 0.070 | [0.014, 0.282] |
| Spouse co-residence | 0.118 | 0.331 | 0.721 | [−0.426, 0.663] |
| ln household income | 0.325** | 0.128 | 0.011 | [0.115, 0.535] |
| Constant | −9.636*** | 3.447 | 0.005 | [−15.307, −3.965] |
| Random-intercept variance (province) | 0.189*** | 0.120 | 0.001 | [0.066, 0.538] |
| LR test (chibar², p) | 9.350, p = 0.001 | | | |
| Obs. | 970 | | | |
| Groups: Provinces | 28 | | | |
| Wald chi2 | 98.890 | | | |
| Log likelihood | −511.921 | | | |

Note:

a: Data are from the China Family Panel Studies (2020); authors’ calculations.

b: *** p < 0.01, ** p < 0.05, * p < 0.10.

c: The model is estimated using two-level mixed-effects logistic regression.

**Table S2.** Association between job quality and short-term fertility intention: two children

| **Variables** | **Coef.** | **Std. Err.** | **p-value** | **90% CI** |
| --- | --- | --- | --- | --- |
| Job quality index | 1.911 | 1.443 | 0.192 | [−0.490, 4.248] |
| Gender | 0.678 | 0.503 | 0.178 | [−0.150, 1.506] |
| Age | −0.662 | 0.409 | 0.105 | [−1.335, 0.010] |
| Age squared | 0.007 | 0.006 | 0.195 | [−0.002, 0.016] |
| Education | 0.294 | 0.235 | 0.211 | [−0.092, 0.681] |
| Education squared | 0.062 | 0.102 | 0.543 | [−0.106, 0.230] |
| Self-rated health | 0.310 | 0.196 | 0.114 | [−0.013, 0.632] |
| Spouse age | 0.081 | 0.080 | 0.313 | [−0.051, 0.213] |
| Spouse education | −0.150 | 0.236 | 0.525 | [−0.538, 0.238] |
| Spouse co-residence | −0.491 | 0.661 | 0.458 | [−1.578, 0.597] |
| ln household income | −0.522 | 0.337 | 0.121 | [−1.075, 0.032] |
| Constant | 10.571 | 7.434 | 0.155 | [−1.657, 22.798] |
| Random-intercept variance (province) | 0.002 | 0.295 | 0.498 | [0.000, +∞] |
| LR test (chibar², p) | 0.000, p = 0.498 | | | |
| Obs. | 918 | | | |
| Groups: Provinces | 27 | | | |
| Wald chi2 | 13.830 | | | |
| Log likelihood | −114.738 | | | |

Note:

a: Data are from the China Family Panel Studies (2020); authors’ calculations.

b: *** p < 0.01, ** p < 0.05, * p < 0.10.

c: The model is estimated using two-level mixed-effects logistic regression.

**Table S3.** Association between job quality and short-term fertility intention: three or more children

| **Variables** | **Coef.** | **Std. Err.** | **p-value** | **90% CI** |
| --- | --- | --- | --- | --- |
| Job quality index | 2.705 | 3.450 | 0.428 | [−2.945, 8.429] |
| Gender | 1.586 | 1.521 | 0.297 | [−0.916, 4.088] |
| Age | 4.168 | 3.243 | 0.199 | [−1.166, 9.502] |
| Age squared | −0.060 | 0.044 | 0.176 | [−0.133, 0.013] |
| Education | −0.535 | 1.096 | 0.625 | [−2.338, 1.267] |
| Education squared | 0.028 | 0.348 | 0.935 | [−0.543, 0.600] |
| Self-rated health | 0.239 | 0.419 | 0.568 | [−0.450, 0.927] |
| Spouse age | 0.246 | 0.194 | 0.206 | [−0.074, 0.565] |
| Spouse education | −0.435 | 0.605 | 0.472 | [−1.429, 0.560] |
| Spouse co-residence | Omitted | — | — | — |
| ln household income | −0.293 | 0.933 | 0.753 | [−1.829, 1.242] |
| Constant | −81.217 | 59.733 | 0.174 | [−179.470, 17.035] |
| Random-intercept variance (province) | 0.000 | 0.000 | — | — |
| LR test (chibar², p) | 0.000, p = NA | | | |
| Obs. | 222 | | | |
| Groups: Provinces | 22 | | | |
| Wald chi2 | 9.700 | | | |
| Log likelihood | −19.505 | | | |

Note:

a: Data are from the China Family Panel Studies (2020); authors’ calculations.

b: *** p < 0.01, ** p < 0.05, * p < 0.10.

c: The model is estimated using two-level mixed-effects logistic regression.

**Table S4.** Association between job quality and short-term fertility intention: control function approach

| **Variables** | **Coef.** | **Std. Err.** | **p-value** | **90% CI** |
| --- | --- | --- | --- | --- |
| Job quality index | −5.782* | 2.988 | 0.054 | [−10.715, −0.846] |
| Residual  (endogeneity correction variable) | 5.131* | 2.994 | 0.058 | [0.148, 10.038] |
| Gender | 0.640*** | 0.186 | 0.001 | [0.333, 0.947] |
| Age | 0.205 | 0.167 | 0.219 | [−0.069, 0.480] |
| Age squared | −0.005* | 0.003 | 0.072 | [−0.009, −0.001] |
| Education | 0.340* | 0.183 | 0.064 | [0.039, 0.642] |
| Education squared | 0.122*** | 0.041 | 0.003 | [0.054, 0.189] |
| Self-rated health | 0.008 | 0.081 | 0.926 | [−0.140, 0.125] |
| Spouse age | −0.042 | 0.028 | 0.143 | [−0.088, 0.005] |
| Spouse education | 0.159* | 0.081 | 0.050 | [0.026, 0.293] |
| Spouse co-residence | 0.324 | 0.311 | 0.296 | [−0.186, 0.835] |
| Parity | −1.780*** | 0.181 | 0.000 | [−2.098, −1.502] |
| ln household income | 0.489** | 0.243 | 0.044 | [0.089, 0.888] |
| Constant | −3.846 | 3.487 | 0.185 | [−10.469, 1.128] |
| Random-intercept variance (province) | 0.168*** | 0.110 | 0.001 | [0.055, 0.487] |
| LR test (chibar², p) | 9.980, p = 0.001 | | | |
| Obs. | 1,979 | | | |
| Groups: Provinces | 27 | | | |
| Wald chi2 | 266.170 | | | |
| Log likelihood | −630.381 | | | |

Note:

a: Data are from the China Family Panel Studies (2020); authors’ calculations.

b: *** p < 0.01, ** p < 0.05, * p < 0.10.

c: The model is estimated using two-level mixed-effects logistic regression.

**Table S5.** Association between job quality and short-term fertility intention: CRITIC-weighted job quality index

| **Variables** | **Coef.** | **Std. Err.** | **p-value** | **90% CI** |
| --- | --- | --- | --- | --- |
| CRITIC-weighted job quality index | −1.087** | 0.516 | 0.037 | [−1.931, −0.231] |
| Gender | 0.563*** | 0.161 | 0.000 | [0.298, 0.827] |
| Age | 0.174 | 0.161 | 0.280 | [−0.091, 0.440] |
| Age squared | −0.004* | 0.002 | 0.085 | [−0.008, −0.001] |
| Education | 0.063 | 0.083 | 0.450 | [−0.074, 0.200] |
| Education squared | 0.078** | 0.039 | 0.048 | [0.013, 0.143] |
| Self-rated health | 0.074 | 0.072 | 0.309 | [−0.045, 0.193] |
| Spouse age | −0.039 | 0.027 | 0.148 | [-0.084, 0.003] |
| Spouse education | 0.118 | 0.074 | 0.110 | [−0.004, 0.240] |
| Spouse co-residence | 0.213 | 0.287 | 0.458 | [−0.260, 0.686] |
| Parity | −1.786*** | 0.175 | 0.000 | [−2.073, −1.499] |
| ln household income | 0.202* | 0.110 | 0.066 | [0.021, 0.382] |
| Constant | −1.807 | 2.771 | 0.438 | [−6.794, 2.437] |
| Random-intercept variance (province) | 0.152*** | 0.098 | 0.001 | [0.051, 0.435] |
| LR test (chibar², p) | 9.370, p = 0.001 | | | |
| Obs. | 2,119 | | | |
| Groups: Provinces | 28 | | | |
| Wald chi2 | 282.560 | | | |
| Log likelihood | −690.375 | | | |

Note:

a: Data are from the China Family Panel Studies (2020); authors’ calculations.

b: *** p < 0.01, ** p < 0.05, * p < 0.10.

c: The model is estimated using two-level mixed-effects logistic regression.

**Table S6.** Association between job quality and short-term fertility intention: age restriction for women under 40

| **Variables** | **Coef.** | **Std. Err.** | **p-value** | **90% CI** |
| --- | --- | --- | --- | --- |
| Job quality index | −0.910* | 0.543 | 0.063 | [−1.823, −0.112] |
| Gender | 0.583*** | 0.165 | 0.000 | [0.312, 0.855] |
| Age | 0.170 | 0.205 | 0.406 | [−0.167, 0.507] |
| Age squared | 0.004 | 0.003 | 0.207 | [−0.009, 0.001] |
| Education | 0.046 | 0.086 | 0.593 | [−0.096, 0.188] |
| Education squared | 0.061 | 0.042 | 0.144 | [−0.008, 0.129] |
| Self-rated health | 0.066 | 0.074 | 0.375 | [−0.056, 0.188] |
| Spouse age | −0.039 | 0.028 | 0.161 | [−0.085, 0.007] |
| Spouse education | 0.144* | 0.076 | 0.057 | [0.020, 0.269] |
| Spouse co-residence | 0.260 | 0.296 | 0.379 | [−0.227, 0.748] |
| Parity | −1.827*** | 0.182 | 0.000 | [−2.126, −1.528] |
| ln household income | 0.265** | 0.116 | 0.023 | [0.073, 0.456] |
| Constant | −1.097 | 3.490 | 0.373 | [−8.479, 2.522] |
| Random-intercept variance (province) | 0.192*** | 0.116 | 0.000 | [0.059, 0.478] |
| LR test (chibar², p) | 11.600, p < 0.001 | | | |
| Obs. | 1,633 | | | |
| Groups: Provinces | 28 | | | |
| Wald chi2 | 241.150 | | | |
| Log likelihood | −652.025 | | | |

Note:

a: Data are from the China Family Panel Studies (2020); authors’ calculations.

b: *** p < 0.01, ** p < 0.05, * p < 0.10.

c: The model is estimated using two-level mixed-effects logistic regression.

**Table S7.** Association between job quality and endorsement of “raising children for old-age support” belief: total sample

| **Variables** | **Coef.** | **Std. Err.** | **p-value** | **90% CI** |
| --- | --- | --- | --- | --- |
| Job quality index | −0.260 | 0.324 | 0.423 | [−0.792, 0.273] |
| Gender | 0.262** | 0.108 | 0.015 | [0.085, 0.440] |
| Age | −0.105 | 0.092 | 0.253 | [−0.256, 0.046] |
| Age squared | 0.002 | 0.001 | 0.198 | [0.000, 0.004] |
| Education | −0.159*** | 0.055 | 0.004 | [−0.249, −0.068] |
| Education squared | 0.044* | 0.023 | 0.056 | [0.006, 0.083] |
| Self-rated health | −0.113** | 0.046 | 0.014 | [−0.189, −0.038] |
| Spouse age | 0.000 | 0.017 | 0.996 | [−0.027, 0.028] |
| Spouse education | −0.148*** | 0.051 | 0.004 | [−0.231, −0.064] |
| Spouse co-residence | 0.089 | 0.162 | 0.584 | [−0.178, 0.355] |
| Parity | 0.134* | 0.070 | 0.057 | [0.018, 0.249] |
| ln household income | −0.094 | 0.071 | 0.185 | [−0.211, 0.023] |
| LR test (chibar², p) | 0.090, p = 0.382 | | | |
| Obs. | 2,119 | | | |
| Groups: Provinces | 28 | | | |
| Wald chi2 | 137.380 | | | |
| Log likelihood | −1981.546 | | | |

Note:

a: Data are from the China Family Panel Studies (2020); authors’ calculations.

b: *** p < 0.01, ** p < 0.05, * p < 0.10.

c: The model is estimated using two-level mixed-effects ordered logistic regression.

**Table S8.** Association between job quality and endorsement of “raising children for old-age support” belief: without male children

| **Variables** | **Coef.** | **Std. Err.** | **p-value** | **90% CI** |
| --- | --- | --- | --- | --- |
| Job quality index | −1.539*** | 0.592 | 0.009 | [−2.512, −0.565] |
| Gender | 0.540*** | 0.193 | 0.005 | [0.223, 0.857] |
| Age | −0.192 | 0.168 | 0.254 | [−0.469, 0.085] |
| Age squared | 0.003 | 0.002 | 0.202 | [−0.001, 0.007] |
| Education | −0.161 | 0.102 | 0.113 | [−0.328, 0.006] |
| Education squared | 0.034 | 0.048 | 0.472 | [−0.044, 0.113] |
| Self-rated health | 0.011 | 0.087 | 0.899 | [−0.132, 0.154] |
| Spouse age | −0.014 | 0.028 | 0.629 | [−0.061, 0.033] |
| Spouse education | −0.090 | 0.090 | 0.320 | [−0.238, 0.059] |
| Spouse co-residence | 0.153 | 0.345 | 0.657 | [−0.414, 0.721] |
| Parity | −0.103 | 0.171 | 0.544 | [−0.384, 0.177] |
| ln household income | −0.117 | 0.128 | 0.362 | [−0.327, 0.094] |
| LR test (chibar², p) | 0.760, p = 0.192 | | | |
| Obs. | 636 | | | |
| Groups: Provinces | 28 | | | |
| Wald chi2 | 57.140 | | | |
| Log likelihood | −596.555 | | | |

Note:

a: Data are from the China Family Panel Studies (2020); authors’ calculations.

b: *** p < 0.01, ** p < 0.05, * p < 0.10.

c: The model is estimated using two-level mixed-effects ordered logistic regression.

**Table S9.** Association between job quality and endorsement of “raising children for old-age support” belief: with male children

| **Variables** | **Coef.** | **Std. Err.** | **p-value** | **90% CI** |
| --- | --- | --- | --- | --- |
| Job quality index | 0.265 | 0.388 | 0.496 | [−0.374, 0.903] |
| Gender | 0.159 | 0.131 | 0.227 | [−0.057, 0.375] |
| Age | −0.085 | 0.114 | 0.456 | [−0.272, 0.102] |
| Age squared | 0.001 | 0.002 | 0.418 | [−0.001, 0.004] |
| Education | −0.145** | 0.068 | 0.032 | [−0.257, −0.034] |
| Education squared | 0.054* | 0.028 | 0.052 | [0.008, 0.100] |
| Self-rated health | −0.155*** | 0.055 | 0.005 | [−0.245, −0.065] |
| Spouse age | 0.008 | 0.021 | 0.707 | [−0.026, 0.042] |
| Spouse education | −0.178*** | 0.062 | 0.004 | [−0.281, −0.075] |
| Spouse co-residence | 0.131 | 0.185 | 0.481 | [−0.174, 0.435] |
| Parity | 0.167** | 0.079 | 0.035 | [0.037, 0.297] |
| ln household income | −0.075 | 0.087 | 0.390 | [−0.217, 0.068] |
| LR test (chibar², p) | 0.000, p = NA | | | |
| Obs. | 1,483 | | | |
| Groups: Provinces | 27 | | | |
| Wald chi2 | 82.250 | | | |
| Log likelihood | −1374.275 | | | |

Note:

a: Data are from the China Family Panel Studies (2020); authors’ calculations.

b: *** p < 0.01, ** p < 0.05, * p < 0.10.

c: The model is estimated using two-level mixed-effects ordered logistic regression.

**Table S10.** Association between job quality and endorsement of “continuing the family lineage” belief: total sample

| **Variables** | **Coef.** | **Std. Err.** | **p-value** | **90% CI** |
| --- | --- | --- | --- | --- |
| Job quality index | −0.687** | 0.322 | 0.033 | [−1.241, −0.184] |
| Gender | 0.765*** | 0.107 | 0.000 | [0.590, 0.941] |
| Age | −0.155* | 0.091 | 0.088 | [−0.305, −0.005] |
| Age squared | 0.002 | 0.001 | 0.189 | [0.000, 0.004] |
| Education | −0.119** | 0.054 | 0.026 | [−0.207, −0.031] |
| Education squared | 0.036 | 0.023 | 0.121 | [−0.002, 0.075] |
| Self-rated health | −0.149*** | 0.045 | 0.001 | [−0.223, −0.074] |
| Spouse age | 0.038** | 0.016 | 0.021 | [0.011, 0.065] |
| Spouse education | −0.153*** | 0.050 | 0.002 | [−0.235, −0.071] |
| Spouse co-residence | 0.096 | 0.160 | 0.550 | [−0.168, 0.359] |
| Parity | 0.197*** | 0.070 | 0.005 | [0.083, 0.312] |
| ln household income | −0.059 | 0.071 | 0.406 | [−0.176, 0.058] |
| LR test (chibar², p) | 8.560, p = 0.002 | | | |
| Obs. | 2,119 | | | |
| Groups: Provinces | 28 | | | |
| Wald chi2 | 187.580 | | | |
| Log likelihood | −2004.230 | | | |

Note:

a: Data are from the China Family Panel Studies (2020); authors’ calculations.

b: *** p < 0.01, ** p < 0.05, * p < 0.10.

c: The model is estimated using two-level mixed-effects ordered logistic regression.

**Table S11.** Association between job quality and endorsement of “continuing the family lineage” belief: without male children

| **Variables** | **Coef.** | **Std. Err.** | **p-value** | **90% CI** |
| --- | --- | --- | --- | --- |
| Job quality index | −1.036* | 0.583 | 0.076 | [−1.995, −0.077] |
| Gender | 0.998*** | 0.190 | 0.000 | [0.684, 1.311] |
| Age | −0.403** | 0.165 | 0.015 | [−0.674, −0.131] |
| Age squared | 0.005** | 0.002 | 0.031 | [0.001, 0.009] |
| Education | −0.056 | 0.097 | 0.565 | [−0.216, 0.104] |
| Education squared | −0.047 | 0.047 | 0.312 | [−0.124, 0.030] |
| Self-rated health | −0.097 | 0.086 | 0.259 | [−0.238, 0.044] |
| Spouse age | 0.052* | 0.028 | 0.063 | [0.006, 0.098] |
| Spouse education | −0.193** | 0.088 | 0.028 | [−0.338, −0.048] |
| Spouse co-residence | −0.205 | 0.338 | 0.545 | [−0.761, 0.352] |
| Parity | −0.238 | 0.165 | 0.151 | [−0.510, 0.034] |
| ln household income | −0.038 | 0.125 | 0.764 | [−0.244, 0.169] |
| LR test (chibar², p) | 6.830, p = 0.005 | | | |
| Obs. | 636 | | | |
| Groups: Provinces | 28 | | | |
| Wald chi2 | 66.050 | | | |
| Log likelihood | −623.272 | | | |

Note:

a: Data are from the China Family Panel Studies (2020); authors’ calculations.

b: *** p < 0.01, ** p < 0.05, * p < 0.10.

c: The model is estimated using two-level mixed-effects ordered logistic regression.

**Table S12.** Association between job quality and endorsement of “continuing the family lineage” belief: with male children

| **Variables** | **Coef.** | **Std. Err.** | **p-value** | **90% CI** |
| --- | --- | --- | --- | --- |
| Job quality index | −0.599 | 0.389 | 0.100 | [−1.280, 0.002] |
| Gender | 0.726*** | 0.131 | 0.000 | [0.510, 0.942] |
| Age | −0.087 | 0.114 | 0.442 | [−0.274, 0.100] |
| Age squared | 0.001 | 0.002 | 0.640 | [−0.002, 0.003] |
| Education | −0.118* | 0.067 | 0.077 | [−0.227, −0.008] |
| Education squared | 0.076*** | 0.028 | 0.007 | [0.030, 0.123] |
| Self-rated health | −0.162*** | 0.055 | 0.003 | [−0.252, −0.073] |
| Spouse age | 0.034* | 0.021 | 0.096 | [0.000, 0.068] |
| Spouse education | −0.117* | 0.062 | 0.058 | [−0.219, −0.015] |
| Spouse co-residence | 0.205 | 0.185 | 0.266 | [−0.098, 0.509] |
| Parity | 0.212*** | 0.080 | 0.008 | [0.080, 0.344] |
| ln household income | −0.043 | 0.088 | 0.621 | [−0.188, 0.101] |
| LR test (chibar², p) | 1.870, p = 0.086 | | | |
| Obs. | 1,483 | | | |
| Groups: Provinces | 27 | | | |
| Wald chi2 | 108.820 | | | |
| Log likelihood | −1358.163 | | | |

Note:

a: Data are from the China Family Panel Studies (2020); authors’ calculations.

b: *** p < 0.01, ** p < 0.05, * p < 0.10.

c: The model is estimated using two-level mixed-effects ordered logistic regression.

**Table S13.** Association between job quality and short-term fertility intention: male migrant workers

| **Variables** | **Coef.** | **Std. Err.** | **p-value** | **90% CI** |
| --- | --- | --- | --- | --- |
| Job quality index | −1.337** | 0.647 | 0.039 | [−2.480, −0.343] |
| Age | 0.160 | 0.197 | 0.416 | [−0.164, 0.484] |
| Age squared | −0.004 | 0.003 | 0.178 | [−0.009, 0.001] |
| Education | 0.126 | 0.106 | 0.232 | [−0.048, 0.300] |
| Education squared | 0.074 | 0.051 | 0.151 | [−0.011, 0.158] |
| Self-rated health | 0.071 | 0.090 | 0.432 | [−0.077, 0.219] |
| Spouse age | −0.032 | 0.034 | 0.344 | [−0.088, 0.024] |
| Spouse education | 0.042 | 0.093 | 0.651 | [−0.111, 0.195] |
| Spouse co-residence | 0.127 | 0.366 | 0.729 | [−0.475, 0.730] |
| Parity | −1.884*** | 0.221 | 0.000 | [−2.248, −1.520] |
| ln household income | 0.258* | 0.145 | 0.076 | [0.019, 0.496] |
| Constant | −1.818 | 3.550 | 0.609 | [−7.657, 4.021] |
| Random-intercept variance (province) | 0.061 | 0.084 | 0.169 | [0.006, 0.590] |
| LR test (chibar², p) | 0.920, p = 0.169 | | | |
| Obs. | 1,235 | | | |
| Groups: Provinces | 27 | | | |
| Wald chi2 | 181.010 | | | |
| Log likelihood | −418.726 | | | |

Note:

a: Data are from the China Family Panel Studies (2020); authors’ calculations.

b: *** p < 0.01, ** p < 0.05, * p < 0.10.

c: The model is estimated using two-level mixed-effects logistic regression.

**Table S14.** Association between job quality and short-term fertility intention: female migrant workers

| **Variables** | **Coef.** | **Std. Err.** | **p-value** | **90% CI** |
| --- | --- | --- | --- | --- |
| Job quality index | 0.152 | 0.833 | 0.851 | [−1.211, 1.524] |
| Age | 0.327 | 0.322 | 0.309 | [−0.202, 0.857] |
| Age squared | −0.007 | 0.005 | 0.166 | [−0.016, 0.001] |
| Education | −0.110 | 0.139 | 0.428 | [−0.338, 0.118] |
| Education squared | 0.093 | 0.060 | 0.123 | [−0.006, 0.192] |
| Self-rated health | 0.062 | 0.124 | 0.619 | [−0.143, 0.267] |
| Spouse age | −0.042 | 0.047 | 0.365 | [−0.119, 0.034] |
| Spouse education | 0.269** | 0.124 | 0.031 | [0.064, 0.473] |
| Spouse co-residence | 0.217 | 0.476 | 0.648 | [−0.565, 0.999] |
| Parity | −1.544*** | 0.288 | 0.000 | [−2.017, −1.070] |
| ln household income | 0.096 | 0.176 | 0.584 | [−0.193, 0.385] |
| Constant | −3.740 | 5.257 | 0.477 | [−12.388, 4.907] |
| Random-intercept variance (province) | 0.171** | 0.146 | 0.030 | [0.042, 0.700] |
| LR test (chibar², p) | 3.550, p = 0.030 | | | |
| Obs. | 884 | | | |
| Groups: Provinces | 28 | | | |
| Wald chi2 | 91.740 | | | |
| Log likelihood | −270.770 | | | |

Note:

a: Data are from the China Family Panel Studies (2020); authors’ calculations.

b: *** p < 0.01, ** p < 0.05, * p < 0.10.

c: The model is estimated using two-level mixed-effects logistic regression.

**Table S15.** Association between job quality and short-term fertility intention: low-income families

| **Variables** | **Coef.** | **Std. Err.** | **p-value** | **90% CI** |
| --- | --- | --- | --- | --- |
| Job quality index | −0.828 | 2.496 | 0.740 | [−4.933, 3.279] |
| Gender | 1.094 | 0.775 | 0.158 | [−0.181, 2.370] |
| Age | −0.090 | 0.536 | 0.866 | [−0.972, 0.791] |
| Age squared | −0.001 | 0.008 | 0.933 | [−0.013, 0.012] |
| Education | −0.350 | 0.443 | 0.430 | [−1.079, 0.379] |
| Education squared | 0.100 | 0.154 | 0.514 | [−0.153, 0.354] |
| Self-rated health | −0.232 | 0.291 | 0.426 | [−0.711, 0.247] |
| Spouse age | 0.085 | 0.096 | 0.377 | [−0.073, 0.242] |
| Spouse education | 0.138 | 0.335 | 0.680 | [−0.413, 0.689] |
| Spouse co-residence | −0.209 | 1.150 | 0.856 | [−2.100, 1.682] |
| Parity | −1.121** | 0.490 | 0.022 | [−1.926, −0.315] |
| ln household income | 0.335 | 0.908 | 0.712 | [−1.158, 1.829] |
| Constant | −2.139 | 12.571 | 0.865 | [−22.817, 18.539] |
| Random-intercept variance (province) | 0.000 | 0.000 | — | — |
| LR test (chibar², p) | 0.000, p = NA | | | |
| Obs. | 265 | | | |
| Groups: Provinces | 23 | | | |
| Wald chi2 | 11.120 | | | |
| Log likelihood | −47.739 | | | |

Note:

a: Data are from the China Family Panel Studies (2020); authors’ calculations.

b: *** p < 0.01, ** p < 0.05, * p < 0.10.

c: The model is estimated using two-level mixed-effects logistic regression.

**Table S16.** Association between job quality and short-term fertility intention: middle-income families

| **Variables** | **Coef.** | **Std. Err.** | **p-value** | **90% CI** |
| --- | --- | --- | --- | --- |
| Job quality index | −1.060* | 0.643 | 0.099 | [−2.184, −0.062] |
| Gender | 0.623*** | 0.215 | 0.004 | [0.269, 0.977] |
| Age | 0.189 | 0.203 | 0.353 | [−0.146, 0.524] |
| Age squared | −0.005 | 0.003 | 0.146 | [−0.010, 0.001] |
| Education | 0.192* | 0.104 | 0.066 | [0.020, 0.364] |
| Education squared | 0.117** | 0.055 | 0.033 | [0.027, 0.206] |
| Self-rated health | 0.069 | 0.093 | 0.462 | [−0.085, 0.222] |
| Spouse age | −0.034 | 0.034 | 0.325 | [−0.090, 0.023] |
| Spouse education | −0.022 | 0.097 | 0.816 | [−0.181, 0.136] |
| Spouse co-residence | −0.382 | 0.352 | 0.278 | [−0.961, 0.198] |
| Parity | −1.737*** | 0.212 | 0.000 | [−2.086, −1.389] |
| ln household income | −0.264 | 0.273 | 0.334 | [−0.713, 0.185] |
| Constant | 2.595 | 4.116 | 0.528 | [−4.176, 9.365] |
| Random-intercept variance (province) | 0.037 | 0.070 | 0.262 | [0.002, 0.819] |
| LR test (chibar², p) | 0.410, p = 0.262 | | | |
| Obs. | 1,344 | | | |
| Groups: Provinces | 27 | | | |
| Wald chi2 | 160.070 | | | |
| Log likelihood | −417.017 | | | |

Note:

a: Data are from the China Family Panel Studies (2020); authors’ calculations.

b: *** p < 0.01, ** p < 0.05, * p < 0.10.

c: The model is estimated using two-level mixed-effects logistic regression.

**Table S17.** Association between job quality and short-term fertility intention: high-income families

| **Variables** | **Coef.** | **Std. Err.** | **p-value** | **90% CI** |
| --- | --- | --- | --- | --- |
| Job quality index | −0.512 | 1.037 | 0.615 | [−2.227, 1.184] |
| Gender | 0.693** | 0.285 | 0.015 | [0.224, 1.161] |
| Age | 0.487 | 0.368 | 0.186 | [−0.118, 1.092] |
| Age squared | −0.009 | 0.006 | 0.123 | [−0.019, 0.001] |
| Education | 0.139 | 0.218 | 0.524 | [−0.220, 0.498] |
| Education squared | −0.114 | 0.100 | 0.254 | [−0.279, 0.050] |
| Self-rated health | 0.138 | 0.146 | 0.344 | [−0.102, 0.378] |
| Spouse age | −0.083 | 0.056 | 0.138 | [−0.175, 0.009] |
| Spouse education | 0.314** | 0.139 | 0.024 | [0.085, 0.544] |
| Spouse co-residence | 1.224* | 0.632 | 0.053 | [0.184, 2.263] |
| Parity | −3.249*** | 0.753 | 0.000 | [−4.486, −2.011] |
| ln household income | 0.296 | 0.252 | 0.240 | [−0.118, 0.711] |
| Constant | −7.809 | 6.672 | 0.242 | [−18.784, 3.166] |
| Random-intercept variance (province) | 0.566*** | 0.373 | 0.002 | [0.191, 1.675] |
| LR test (chibar², p) | 8.710, p = 0.002 | | | |
| Obs. | 510 | | | |
| Groups: Provinces | 27 | | | |
| Wald chi2 | 63.280 | | | |
| Log likelihood | −203.468 | | | |

Note:

a: Data are from the China Family Panel Studies (2020); authors’ calculations.

b: *** p < 0.01, ** p < 0.05, * p < 0.10.

c: The model is estimated using two-level mixed-effects logistic regression.
