# Supplementary material for: Job quality and fertility intentions among Chinese migrant workers: the role of traditional fertility beliefs
Source: Front Psychol. 2026 Jun 17;17:1739790. doi: 10.3389/fpsyg.2026.1739790 (PMC13320662; doi:10.3389/fpsyg.2026.1739790)
Supplement: Supplementary file 2 [file Supplementary_file_1.docx]

# Appendix

**Table A1.** Descriptive statistics and correlation matrix for six job quality dimensions

| **Dimension** | **Mean** | **Std. Dev.** | **1** | **2** | **3** | **4** | **5** | **6** |
| --- | --- | --- | --- | --- | --- | --- | --- | --- |
| 1. Wage Income | 2.72 | 0.83 | 1.000 |  |  |  |  |  |
| 2. Job Stability | 0.68 | 0.67 | 0.244* | 1.000 |  |  |  |  |
| 3. Work Intensity | 55.86 | 17.35 | 0.389* | 0.136* | 1.000 |  |  |  |
| 4. Welfare Protection | 0.38 | 0.45 | 0.374* | 0.472* | 0.230* | 1.000 |  |  |
| 5. Career Development Prospects | 0.65 | 0.48 | 0.185* | 0.240* | 0.156* | 0.316* | 1.000 |  |
| 6. Job Satisfaction | 3.33 | 0.80 | 0.222* | 0.181* | 0.292* | 0.231* | 0.541* | 1.000 |

Note:

a: Data are from the China Family Panel Studies (2020); authors’ calculations.

b: *** p < 0.01, ** p < 0.05, * p < 0.10.

c: N = 2,119; Pearson correlation coefficients are reported below the diagonal.

d: Wage income = natural logarithm of hourly wages; Welfare protection = mean of five social insurance indicators; Job satisfaction = mean of five satisfaction indicators; the remaining dimensions are measured using single indicators.

**Table A2.** Characteristics of the study sample and the full eligible CFPS sample of migrant workers

| **Characteristics** | **Study sample (N = 2,119)** | **Full eligible sample (N = 2,422)** |
| --- | --- | --- |
| **Gender** |  |  |
| –Male (%) | 58.28 | 58.08 |
| –Female (%) | 41.72 | 41.92 |
| **Age** |  |  |
| –≤30 (%) | 28.79 | 29.31 |
| –31-35 (%) | 33.93 | 32.87 |
| –36-40 (%) | 20.06 | 20.31 |
| –41-45 (%) | 14.82 | 15.03 |
| –46-49 (%) | 2.41 | 2.48 |
| **Education level** |  |  |
| –Junior high school or below (%) | 52.24 | 53.84 |
| –High School/Associate degree (%) | 37.24 | 36.09 |
| –Bachelor or above (%) | 10.52 | 10.07 |
| **Number of children (Parity)** |  |  |
| –0 children (%) | 0.42 | 0.87 |
| –1 child (%) | 45.78 | 44.80 |
| –2 children (%) | 43.32 | 43.19 |
| –≥3 children (%) | 10.48 | 11.15 |

Note:

a: Data are from the China Family Panel Studies (2020); authors’ calculations.

b: “Full eligible sample” refers to all respondents in the CFPS (2020) who meet the official definition of migrant workers (agricultural hukou holders engaged in non-agricultural industries for six months or more), are employed in wage-earning jobs, are currently active in the labor market, have valid fertility intention data, are currently married with a living spouse, and meet the reproductive age criteria (female respondents aged ≤44 or male respondents with spouses aged ≤44). Ages above 44 therefore occur only among male respondents.

c: The study sample is further restricted to respondents with complete data on key variables.
